# Novel Targets of the CbrAB/Crc Carbon Catabolite Control System Revealed by Transcript Abundance in *Pseudomonas aeruginosa*

**DOI:** 10.1371/journal.pone.0044637

**Published:** 2012-10-24

**Authors:** Elisabeth Sonnleitner, Martina Valentini, Nicolas Wenner, Feth el Zahar Haichar, Dieter Haas, Karine Lapouge

**Affiliations:** 1 Max F. Perutz Laboratories, Department of Microbiology, Immunobiology and Genetics, University of Vienna, Dr. Bohrgasse 9, Vienna, Austria; 2 Department of Fundamental Microbiology, University of Lausanne, Lausanne, Switzerland; Vrije Universiteit Brussel, Belgium

## Abstract

The opportunistic human pathogen *Pseudomonas aeruginosa* is able to utilize a wide range of carbon and nitrogen compounds, allowing it to grow in vastly different environments. The uptake and catabolism of growth substrates are organized hierarchically by a mechanism termed catabolite repression control (Crc) whereby the Crc protein establishes translational repression of target mRNAs at CA (catabolite activity) motifs present in target mRNAs near ribosome binding sites. Poor carbon sources lead to activation of the CbrAB two-component system, which induces transcription of the small RNA (sRNA) CrcZ. This sRNA relieves Crc-mediated repression of target mRNAs. In this study, we have identified novel targets of the CbrAB/Crc system in *P. aeruginosa* using transcriptome analysis in combination with a search for CA motifs. We characterized four target genes involved in the uptake and utilization of less preferred carbon sources: *estA* (secreted esterase), *acsA* (acetyl-CoA synthetase), *bkdR* (regulator of branched-chain amino acid catabolism) and *aroP2* (aromatic amino acid uptake protein). Evidence for regulation by CbrAB, CrcZ and Crc was obtained *in vivo* using appropriate reporter fusions, in which mutation of the CA motif resulted in loss of catabolite repression. CbrB and CrcZ were important for growth of *P. aeruginosa* in cystic fibrosis (CF) sputum medium, suggesting that the CbrAB/Crc system may act as an important regulator during chronic infection of the CF lung.

## Introduction

*Pseudomonas aeruginosa* is a metabolically versatile organism, which is able to grow in a variety of different ecological niches. Its natural habitats can be nutrient-poor (e.g., aqueous environments) or nutrient-rich (e.g., the rhizosphere or infected animal tissues). The CbrAB/Crc carbon catabolite control system helps to ensure a metabolic optimization process by allowing preferential utilization of good energy sources and establishing a healthy carbon (C) to nitrogen (N) balance [1]. The CbrAB/Crc system involves a regulatory protein (Crc), which is required to establish translational repression of target mRNAs at CA (catabolite activity) motifs, usually located in the vicinity of ribosome binding sites [1]–[4]. The core sequence of the CA motif consists of eight nucleotides, AAnAAnAA, where n stands for any nucleotide. However, inspection of RNAs that are under Crc control suggests that n = C or U may be preferred over n = A or G in CA motifs [1], [2]. A small RNA (sRNA) termed CrcZ, which possesses several conserved CA motifs and appears to bind to Crc, antagonizes translational repression mediated by the Crc protein [1]. CrcZ levels are low in the presence of a good carbon source (e.g., succinate), elevated in the presence of an intermediate carbon source (e.g., glucose) and high when a less preferred carbon compound is used as the sole energy source (e.g., mannitol). This regulation is established by the activity of the two-component system CbrAB consisting of the membrane-bound sensor CbrA and the response regulator CbrB, which are required for the expression of *crcZ*, together with the alternative sigma factor RpoN and IHF protein [1], [5]. CbrB binds to an upstream activating sequence (UAS) in the *crcZ* promoter region [5]. Mutants affected in *cbrB* or *crcZ* have similar, but not identical phenotypes, indicating that CrcZ mediates most, but not all, output of the CbrAB system [1], [5]. The CbrAB/Crc system is involved in the regulation of swarming, biofilm formation, cytotoxicity and antibiotic resistance in *P. aeruginosa* [6], [7]. However, in these interactions the regulatory mechanisms have not been investigated in molecular detail.

While the effects of the CbrAB/Crc system occur essentially at a post-transcriptional level, we asked whether transcript abundance could be used to reveal targets of this system. Previous experience with the mechanistically similar GacSA/RsmA pathway in *P. aeruginosa* [8]–[10] and *P. fluorescens* Pf-5 [11] suggests that during translational repression transcript abundance tends to be low, whereas transcript levels may be elevated during derepression, presumably reflecting effects of translational control on mRNA stability. Based on these observations, we decided to compare the transcriptome of the *P. aeruginosa* wild type PAO1 with that of its *crc*, *crcZ* and *cbrB* mutants. To find new targets of the CbrAB/Crc system, we focussed on several mRNAs having appropriately located CA motifs. The targets chosen are all involved in the utilization of relatively poor C sources. Furthermore, we found that growth of *cbrB* and *crcZ* mutants was severely impaired in an artificial cystic fibrosis (CF) sputum medium, an effect that is most likely due to repression of amino acid uptake and catabolism by the Crc protein. Thus, the CbrAB/Crc system may be an important regulatory system enabling *P. aeruginosa* to thrive in the CF lung.

## Results

*Transcriptome analysis of genes regulated by Crc –* To assess the impact of the CbrAB/Crc pathway on global gene expression of *P. aeruginosa*, we performed a transcriptome analysis of *P. aeruginosa* PAO1 wild type and its *crc*, *crcZ* and *cbrB* deletion mutants using commercially available genome-wide DNA microarrays (Affymetrix). First, we grew cells to late exponential phase in Luria Broth (LB) as a nutrient rich medium, which mainly contains amino acids, peptides and small amounts of sugars and nucleotides [12] and which creates conditions of moderate carbon catabolite repression [3]. Second, we used a defined basal salts medium (BSM) amended with 40 mM succinate, which establishes strong catabolite repression in strain PAO1 [1]. Under both conditions, Crc mediates catabolite repression of *amiE*, which encodes short-chain aliphatic amidase and has previously served as a reporter gene ([1]; Figure S1). We expected that genes whose expression is translationally repressed by the CbrAB/Crc system might show elevated transcript levels in a *crc* mutant, whereas down-regulated transcript levels might be seen in both *cbrB* and *crcZ* mutants, due arrest of translation.

**Figure 1:**
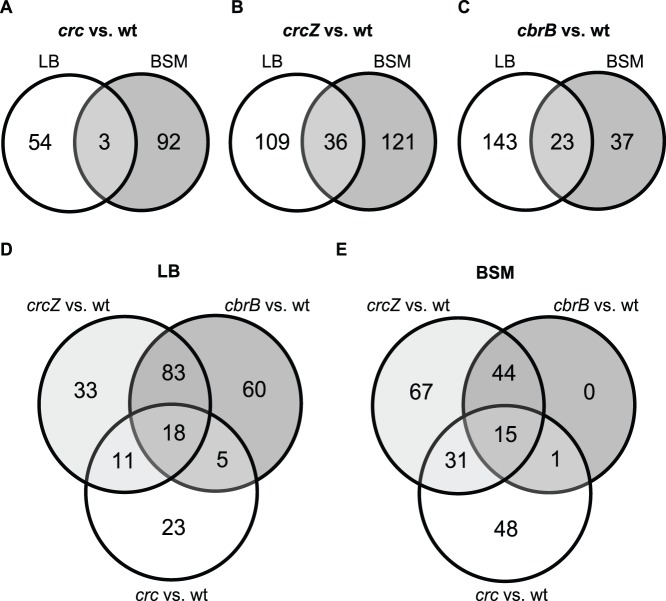
Venn diagrams summarizing changes in transcript abundance in mutants affected in the CbrAB/Crc cascade. Data are taken from Tables S1 and S2. Changes in transcript levels from LB *vs.* BSM-succinate cultures are shown for (**A**) PAO1Δ*crc*, (**B**) PAO1Δ*crcZ* and (**C**) PAO1Δ*cbrB*, by comparison with the wild-type strain. Overlaps between the differentially regulated transcripts in the *crc*, *crcZ* and *cbrB* mutants are shown (**D**) for LB cultures and (**E**) for BSM-succinate cultures.

In LB-grown cells, we found 57 transcripts to be differentially (≥2.0-fold) expressed in the *crc* mutant, by comparison with the wild type PAO1; 19 transcripts were up-regulated and 38 were down-regulated at least two-fold (Table S1). After growth in BSM containing succinate, 95 transcripts showed altered expression; 53 transcripts were up-regulated and 42 were down-regulated (Table S1). The overlap of genes that were differentially affected under both conditions was small (Figure 1A). The probable reason is that transcriptional expression of catabolic genes varies greatly between the two media, making it difficult to detect the same effects of Crc on transcript abundance. Nevertheless, the combined data from both media enabled us to see a considerable number of transcripts influenced by Crc. As a control, we checked *amiE* transcript levels. They were elevated in the Δ*crc* mutant cultivated in LB (Table 1 and Table S1), which is consistent with increased *amiE* translation (Figure S1) and, presumably, with concomitantly enhanced *amiE* mRNA stability in the absence of Crc-mediated catabolite repression. By contrast, the Δ*crc* mutation had no effect on *amiE* transcript abundance in cells grown in BSM, which lacks inducing amides and hence does not permit transcriptional expression of the *amiE* operon [1], [13]. These results suggest that transcriptome analysis may be used to identify targets of the CbrAB/Crc system provided that sufficient transcriptional expression of the target genes takes place.

**Table 1:** Selection of targets of the CbrAB/Crc system implicated by the transcriptome analysis.

ORF^a^	Gene	LB^b^			BSM^b^ + succinate			Description	CA motif (location)^c^
		*crc vs.* *wt*	*cbr vs.* *wt*	*crcZ vs.* *wt*	*crc vs.* *wt*	*cbrB vs.* *wt*	*crcZ vs.* *wt*		
PA0755	*opdH*	5,9			2,05			*cis*-aconitate porin OpdH	AAGAACAA (−25 to −18)
PA0866	***aroP2***	4,73						aromatic amino acid transport protein	AACAAUAA (−33 to −12)
PA0887	***acsA***	−3,41	−2,51	−3,27	7,98	2,88	3,62	acetyl-coenzyme A synthetase	AACAAAAACAA (−35 to −25)
PA0996	*pqsA*		−3,55	−3,49	−2,01	−18,34	−16,67	probable coenzyme A ligase	
PA0997	*pqsB*		−3,72	−3,49	−2,11	−33,37	−56,5	PqsB protein	
PA0998	*pqsC*		−3,73	−3,89	−2,01	−25,21	−40,64	PqsC protein	
PA0999	*pqsD*		−3,77	−3,38	−2	−23,39	−34,65	3-oxoacyl-[acyl-carrier-protein] synthase III	
PA1000	*pqsE*		−2,79	−2,58	−2,09	−18,62	−22,18	quinolone signal response protein	
PA1001	*phnA*		−3,58	−3,28	−2,08	−18,69	−23,34	anthranilate synthase component I	
PA1002	*phnB*		−2,04		−2,01	−11,69	−11,46	anthranilate synthase component II	
PA1003	*pqsR*						−2,48	transcriptional regulator PqsR (MvfR)	
PA1070	*braG*		−2,28	−3,76			−3,22	branched-chain amino acid transport protein BraG	
PA1071	*braF*		−2,37	−4,02	2,08		−2,91	branched-chain amino acid transport protein BraF	
PA1072	*braE*		−2,21	−3,11	2,33		−3,05	branched-chain amino acid transport protein BraE	
PA1073	*braD*		−2,88		2,5		−5,56	branched-chain amino acid transport protein BraD	
PA1074	*braC*		−2,11	−3,6	2,14		−2,41	branched-chain amino acid transport protein BraC	
PA1342		−2,68		−2,38				probable binding protein component ofABC transporter	AAUAAAAA (−33 to −26)
PA1617					2,44			probable AMP-binding enzyme	AACAACAACAA (−23 to −13)
PA1894					5,83		−4,24	hypothetical protein	
PA1895					4,27		−3,07	hypothetical protein	
PA1984	*exaC*		−2,76		3,84		−3,37	NAD+ dependent aldehyde dehydrogenase ExaC	
PA1985	*pqqA*		11,34	8,74				pyrroloquinoline quinone biosynthesis protein A	
PA1986	*pqqB*		2,47	2,52				pyrroloquinoline quinone biosynthesis protein B	
PA1987	*pqqC*		3,86	3,7				pyrroloquinoline quinone biosynthesis protein C	
PA1988	*pqqD*		3,7	4,02				pyrroloquinoline quinone biosynthesis protein D	
PA1989	*pqqE*		2,72	2,77				pyrroloquinoline quinone biosynthesis protein E	
PA2247	***bkdA1***		−2,36	−3,81	3,23			2-oxoisovalerate dehydrogenase (alpha subunit)	
PA2248	*bkdA2*		−2,6	−3,51				2-oxoisovalerate dehydrogenase (beta subunit)	
PA2249	*bkdB*		−2,48	−3,22				branched-chain alpha-keto acid dehydrogenase	
PA2250	*lpdV*		−2,62	−3,07				lipoamide dehydrogenase-Val	
PA2533					2,13			probable sodium:alanine symporter	AACAAGAAUAA (−20 to −10)
PA3038			−4,13		16,75	2,11		probable porin	AAUAACAA (−7 to +1)
PA3190		−7,43		−7,81				probable binding protein component ofABC sugar transporter	AAUAACAA (−24 to −17)
PA3366	***amiE***	4,65	2,04					aliphatic amidase	AACAACAA (−20 to −13)
PA3452	*mqoA*	4,87	3,12					malate:quinone oxidoreductase	
PA3570	*mmsA*				2,23			methylmalonate-semialdehyde dehydrogenase	AACAAUAA (−37 to −30)
PA3875	*narG*	−6,01	−4,46	−4,79				respiratory nitrate reductase alpha chain	AAGAAGAA (+34 to +41)
PA4139					−3,37	2,87	4,72	hypothetical protein	
PA4147	*acoR*				3,38			transcriptional regulator AcoR	AACAACAA (−30 to −23)
PA4150	*acoA*				2,77			probable dehydrogenase E1 component	AACAACAA (−9 to −2)
PA4151	*acoB*				4,1			acetoin catabolism protein AcoB	AACAAGAA (−22 to −15)
PA4306	*flp*				−2,9	−6,66	−8,64	Type IVb pilin, Flp	AACAAGAA (−22 to −15)
PA4496			−2,54	−2,55	2,9	−2,73	−8,18	probable binding protein component of ABC transporter	
PA4500			−3,29	−3,94	2,03			probable binding protein component of ABC transporter	AAAAAGAAAAAA (−22 to −11)
PA4501	*opdD*		−2,53		2,64			glycine-glutamate dipeptide porin OpdP	AACAAUAA (−37 to −30)
PA4770	*lldP*				2,96			L-lactate permease	AACAACAA (−25 to −18)
PA4913					6,89		−2,07	probable binding protein component of ABC transporter	AACAACAA (−53 to −46)
PA5112	***estA***			−2,25	2		−2,23	esterase EstA	AAAAACAA (−24 to −17)
PA5153			−2.42	−2.55	2.92		−2.28	probable periplasmic binding protein	
PA5167	*dctP*		−4,35	−4,05	3,37		−6,85	probable c4-dicarboxylate-binding protein	AAGAACAA (−20 to −13)
PA5168	*dctQ*		−2,19	−2,12	6,54		−6,4	probable dicarboxylate transporter	AAUAAGAA (−20 to −13)
PA5169	*dctM*		−2,41	−2,2	6,72		−10,69	probable C4-dicarboxylate transporter	
PA5220					2,38		−4,08	hypothetical protein	AAGAACAACAAGAA (−31 to −18)
PA5348					5,54		−3,03	probable DNA-binding protein	AACAACAA (−26 to −19)

a The numbers represents ORF according to www. pseudomonas.com. [45].

b Fold changes observed after growth in LB and BSM + succinate, respectively. Positive values indicate that transcripts were more abundant in the mutant than in the wild type. Negative values indicate that transcripts were less abundant in the mutant than in the wild type.

c The locations of the CA-motif are given according to the start codon (A of the ATG = +1).

Transcriptome analysis may reveal both and indirect effects of the CbrAB/Crc system. Direct effects are assumed to involve at least one CA motif (AAnAAnAA) in the region of −50 to +50 nucleotides relative to the translational start site in target mRNAs. We found 29 Crc-regulated transcripts having an appropriately located putative CA motif (Table S1). With the exception of four transcripts (three of them in LB and one in BSM), all mRNAs harbouring a CA motif were up-regulated in the *crc* mutant.

*Transcriptome analysis of crcZ and cbrB mutants -* As mentioned above, the Crc protein and the CrcZ sRNA, which requires CbrB to be transcribed, have opposite regulatory effects; thus one might expect that transcripts that are up-regulated in a *crc* mutant would be down-regulated in *crcZ* and *cbrB* mutants and vice versa. To investigate this assumption, we performed transcriptome analyses with a *crcZ* and a *cbrB* mutant under the same conditions as above. In LB-grown cells, 145 transcripts were differentially expressed by a factor of ≥2.0 in PAO1Δ*crcZ*, whereas 166 transcripts were differentially expressed in PAO1Δ*cbrB*, by comparison with the wild type PAO1 (Table S2). In BSM-grown cells, 157 transcripts showed differential regulation in the *crcZ* mutant and 60 transcripts in the *cbrB* mutant (Table S2). Again, the number of genes showing a similar expression pattern in both media was low (Figure 1B and 1C). However, the *crcZ* and *cbrB* mutations had largely parallel effects; both mutations affected 101 out of 210 transcripts and 59 out of 158 transcripts in LB (Figure 1D) and in BSM (Figure 1E), respectively. Among the transcripts that were affected by both mutations, a majority was down-regulated (Table S2). Some transcripts seemed to be only regulated by CbrB and not by CrcZ. However, preliminary attempts to identify CbrB binding sites upstream of the corresponding ORFs have not been successful. Apart from the *crcZ* promoter, known direct CbrB targets include the *lipA* and *hutU* promoters [5], [14]. However, these genes did not appear in our transcriptome analysis, presumably because of lack of transcriptional inducers in the growth media. Some transcripts whose abundance appeared to be only regulated by CrcZ, but not by CbrB, were also seen, but were considered as questionable since *crcZ* expression strictly depends on CbrB [1]. Taken together, our transcriptome analysis is consistent with the current model stating that the sRNA CrcZ mediates most effects of the CbrAB two-component system.

Somewhat unexpectedly, the overlap between transcripts whose abundance was affected by Crc as well as by CrcZ and/or CbrB in an opposite manner was low in BSM-grown cells (17 out of 47 genes; Figures 1D and 1E, Table 1) and absent in LB-grown cells. Among the genes that did show the anticipated transcript pattern were *estA* (encoding a secreted esterase), the *dctPMQ* operon (required for C_4_-dicarboxylate transport at low concentrations), *braC* and the *braDEFG* operon (which are important for transport of branched chain amino acids) (Table 1). Moreover, *estA, dctP* and *braC* possess CA motifs indicating that they may be targets of the CbrAB/Crc system.

Transcripts that were strongly up-regulated in LB in the absence of Crc, but not down-regulated in the absence of CrcZ or CbrB, included *opdH* (encoding a *cis*-aconitate porin) and *aroP2* (encoding an aromatic amino acid transporter) (Table 1). For *opdH* and *aroP2* candidate CA motifs were found. Transcripts that were strongly up-regulated in BSM in the absence of Crc included *acsA* (encoding acetyl-CoA synthetase required for acetate catabolism), *opdH*, *braC*, *braDEFG*, *bkdA1* (first gene of an operon involved in branched-chain amino acid assimilation), *acoR* and *acoAB* (for acetoin catabolism). CA motifs were found in all of these transcripts except for *braDEFG* and *bkdA1* (Table 1), which provides evidence that these genes may be targets of the CbrAB/Crc system.

Some striking effects of the CbrAB/Crc system on the transcriptome were not associated with CA motifs. For example, the *pqsABCDE* and *phnAB* operons were strongly down-regulated in the *crcZ* and *cbrB* mutants, especially in minimal medium with succinate (Table 1). Both operons are required for the biosynthesis of the *Pseudomonas* quinolone signal (PQS) and 2-heptyl-4-hydroxyquinoline, which act as quorum sensing signal molecules together with the transcriptional regulator PqsR [15]. Transcript abundance of *pqsR* was also affected, but to a lesser extent (Table 1). As none of these genes showed elevated transcript levels in the *crc* mutant, their regulation by the CbrAB/Crc system is probably indirect. Moreover, several genes and operons involved in the anaerobic denitrification pathway were affected by the CbrAB/Crc system at the transcript level (Tables S1 and S2), although the cells had been grown aerobically. However, except for *narG*, which has a poorly conserved CA motif (Table 1), none of the corresponding transcripts showed a CA motif, suggesting indirect regulation by CbrAB/Crc. Finally, in cells cultivated in LB, *pqqA* (encoding the pyrroloquinoline quinone [PQQ] biosynthesis protein A) was one of the most strongly up-regulated transcripts in the *crcZ* and *cbrB* mutants (Table 1). *P. aeruginosa* when growing on ethanol uses a PQQ-dependent ethanol oxidation system (encoded by the *exaA* and *exaBC* genes), which converts ethanol to acetate. Acetyl-CoA synthetase (encoded by the *acsA* gene) is also essential for ethanol utilization [16]. The transcripts of the *pqqABCDE* operon and of the *acsA* and *exaC* genes were all differentially expressed in LB when either *crcZ* or *cbrB* was mutated (Table 1). However, as mentioned above, only the *acsA* transcript was also affected by a *crc* mutation. Thus, regulation of PQQ biosynthesis by the CbrAB/Crc system appears to be mostly indirect. The *agmR* gene encoding a positive regulator of PQQ biosynthesis [17] is a good candidate as it exhibits a CA motif (AUAACGACAAUAA).

*Validation of candidate targets -* In the remainder of this study, we will focus on transcripts (*estA*, *acsA*, and *aroP2*) that have increased abundance in a *crc* mutant and all contain a putative CA motif (i.e. AAnAAnAA). In addition, we investigated the role of the CbrAB/Crc system on *bkdR* and bkdA1, as the *bkdA1* transcript was highly upregulated in the *crc* mutant and the cognate regulatory gene *bkdR* contains a putative CA-motif in the vicinity of its ribosome binding site. All these genes are involved in the utilization of less preferred carbon sources. Our initial concern was to validate the microarray results by an independent method. Data obtained by RT-qPCR (Table 2) were in full agreement with the microarray data (Table 1). In particular, the RT-qPCR measurements obtained in LB-grown cells proved to be more sensitive and hence possibly more accurate than the microarray data.

**Figure 2:**
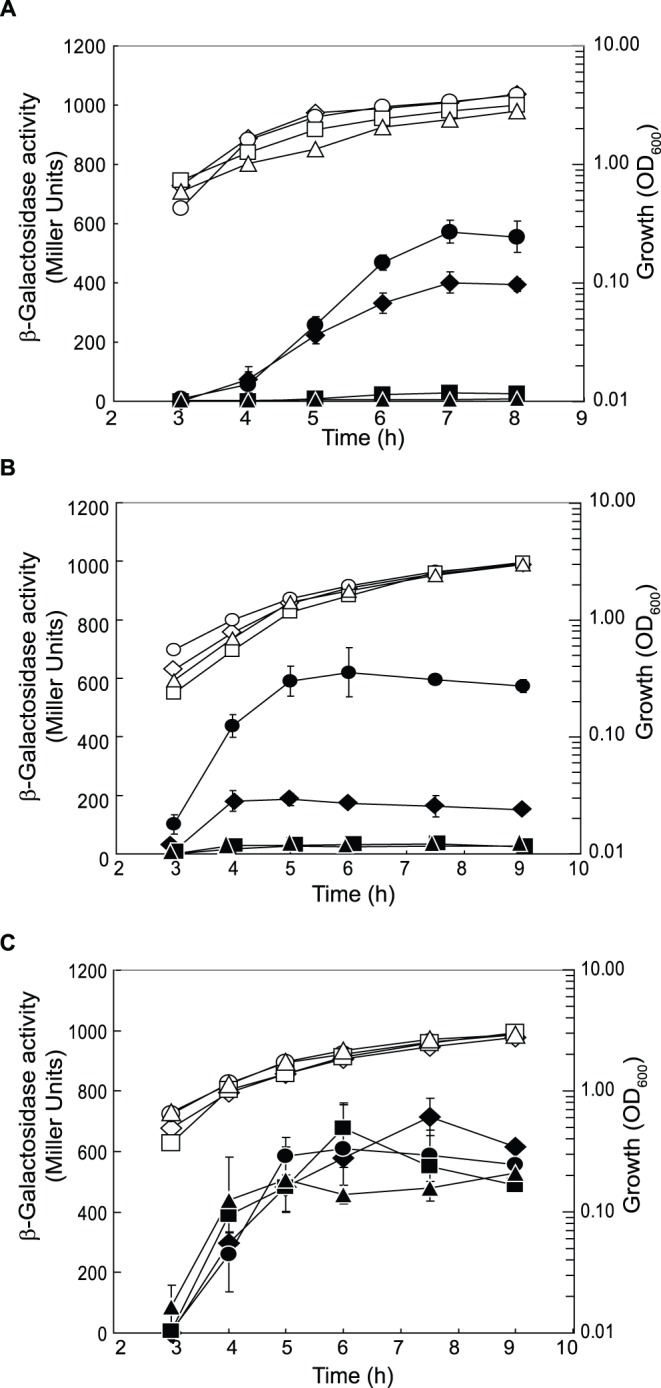
EstA esterase is a target of the CbrAB/Crc cascade. β-Galactosidase activities of an *estA’-‘lacZ* fusion were measured in PAO1 (black diamonds), PAO1Δ*cbrB* (black triangles), PAO1Δ*crcZ* (black squares) and PAO1Δ*crc* (black circles) harbouring a plasmid with a translational *estA’-‘lacZ* fusion (pTLestA) (**A**) in LB medium and (**B**) in BSM medium supplemented with 40 mM succinate. (**C**) β-Galactosidase activities derived from a translational *estA’-‘lacZ* fusion with a mutated CA motif (pTLestA-ΔCA) were determined in PAO1 (black diamonds), PAO1Δ*cbrB* (black triangles), PAO1Δ*crcZ* (black squares) and PAO1Δ*crc* (black circles). The strains were grown in BSM supplemented with 40 mM succinate. Cell growth was monitored by measuring the optical density at 600 nm (OD_600_) (white symbols).

*Regulation of uptake and degradation of poor carbon sources -* Previously, only *amiE* and *phzM* mRNAs have been characterized as targets of the CbrAB/Crc system in *P. aeruginosa* [1], [18]. In this section, we will provide evidence that *estA* and *acsA* are also targets. EstA esterase is an autotransporter that possesses lipolytic enzyme activity [19] and contributes to the hydrolysis of glycerol esters with short-chain fatty acids, which can be further used as carbon sources by *P. aeruginosa* [20]. EstA is located in the outer membrane [21]. In our RT-qPCR analysis, *estA* mRNA appeared to be down-regulated in PAO1Δ*crcZ* and PAO1Δ*cbrB* and up-regulated in PAO1Δ*crc* grown under both growth conditions (Table 2). A translational *estA’-‘lacZ* fusion was assayed for β-galactosidase activity in strain PAO1 and in the *cbrB*, *crcZ* and *crc* mutants, after growth in LB (Figure 2A) and BSM supplemented with 40 mM succinate as the sole carbon source (Figure 2B). Under both conditions, *estA’-‘lacZ* expression was repressed in PAO1Δ*crcZ* and PAO1Δ*cbrB* and derepressed in PAO1Δ*crc*. However, the *estA’-‘lacZ* expression profiles showed quantitative differences in LB (Figure 2A) *vs.* BSM-succinate (Figure 2B), in agreement with the transcript analysis (Tables 1 and 2). To confirm that *estA* is a target of the CbrAB/Crc system we mutated the CA motif, which is located 17 nucleotides upstream of the *estA* start codon (Table 1). In the absence of the wild-type CA motif, the CbrAB/Crc system lost its ability to regulate *estA* completely (Figure 2C).

**Table 2:** Fold changes of transcript abundance in Δ*crc*, Δ*crcZ* and Δ*cbrB* mutants compared to wild type PAO1 in LB and BSM + succinate as determined by RT-qPCR.

Gene	LB^a^			BSM^a^ + succinate		
	*crc vs.* *wt*	*cbr vs.* *wt*	*crcZ vs.* *wt*	*crc vs.* *wt*	*cbr vs.* *wt*	*crcZ vs.* *wt*
*estA*	2.6±0.4	−26.3±0.3	−4.9±0.3	2.5±0.4	−4.1±0.2	−7.2±0.1
*acsA*	4.3±0.2	−10.1±0.7	−7.5±0.1	7.5±0.2	−1.1±0.4	1.5±1.1
*bkdR*	2.3±0.4	−1.7±0.9	1.5±0.9	1.4±1.0	1.5±1.0	1.1±0.8
*bkdA1*	4.0±0.2	−25.4±0.2	−12.3±0.1	9.3±0.1	−3.0±0.3	−2.3±0.2
*ilvE*	3.2±0.2	1.4±0.2	1.8±0.6	1.2±0.9	2.3±1.2	−1.2±0.5
*aroP2*	2.7±0.4	−8.4±1.4	−2.4±0.6	−1.4±1.0	1.0±0.9	1.4±0.9

a Values are represented as averages of 3 independent replicates for every strain.

**Figure 3:**
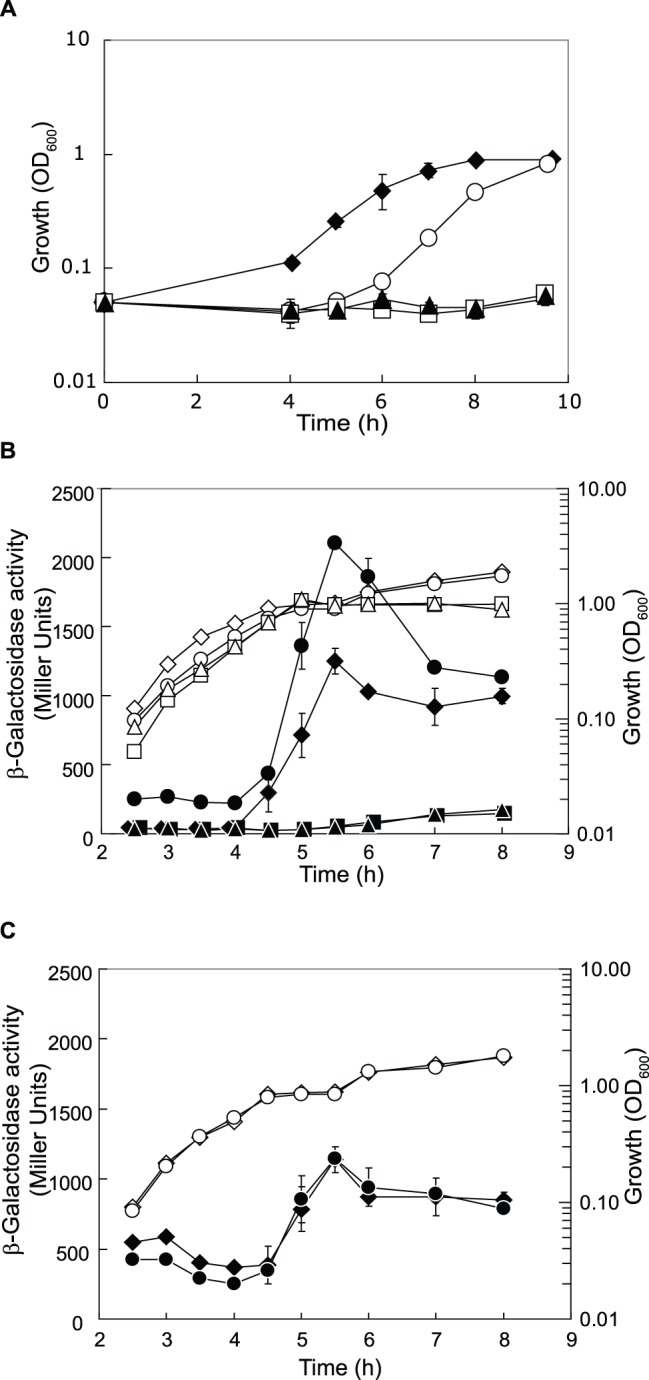
Growth on acetate and *acsA* expression are under CbrAB/Crc control. (**A**) Growth of PAO1 (black diamonds), PAO1Δ*cbrB* (black triangles), PAO1Δ*crcZ* (white squares) and PAO1Δ*crc* (white circles) was measured in BSM supplemented with 40 mM acetate. (**B**) β-Galactosidase expression of a translational *acsA’-‘lacZ* fusion (pME10044) was monitored in PAO1 (black diamonds), PAO1Δ*cbrB* (black triangles), PAO1Δ*crcZ* (black squares) and PAO1Δ*crc* (black circles). (**C**) Expression of a translational *acsA’-‘lacZ* fusion where the CA motif had been mutated (pME10045) was followed in PAO1 (black diamonds) and PAO1Δ*crc* (black circles). Cells were grown in BSM supplemented with 5 mM succinate and 40 mM acetate. The corresponding growth curves are shown in white symbols.

*P. aeruginosa* can use acetate as a sole carbon and energy source, by converting acetate to acetyl-CoA, which is further metabolized via the tricarboxylic acid and glyoxylic acid cycles to generate energy and cell components. The initial reaction is catalyzed by acetyl-CoA synthetase, the product of the *acsA* gene. Acetyl-CoA synthetase activity is induced during growth on acetate or on ethanol [16], [22]. In our transcript analysis, *acsA* was differentially expressed in the wild type PAO1 and the *crc*, *crcZ* and *cbrB* mutants (Tables 1 and 2). The unexpected and unexplained finding here is that the *cbrB* and *crcZ* mutants on the one hand and the *crc* mutant on the other did not show opposite effects on *ascA* expression (Table 1). To demonstrate that the CbrAB/Crc system regulates *acsA*, we measured the β-galactosidase activity of a translational *acsA’-‘lacZ* fusion in these strains. As strain PAO1 lacking *crcZ* or *cbrB* showed a severe growth defect on acetate as the sole carbon source (Figure 3A), we grew the cells in BSM supplemented with 40 mM acetate and 5 mM succinate. As expected, *ascA’-‘lacZ* expression was completely repressed in PAO1Δ*crcZ* and PAO1Δ*cbrB*, and increased in PAO1Δ*crc*, by comparison with PAO1 (Figure 3B). The *acsA* mRNA harbours an extended CA motif (AACAAAAACAA) located 25 nucleotides upstream of the *acsA* start codon. Mutation of the CA motif in the *acsA* leader of an *acsA’-‘lacZ* construct resulted in loss of catabolite repression mediated by Crc (Figure 3C).

**Figure 4:**
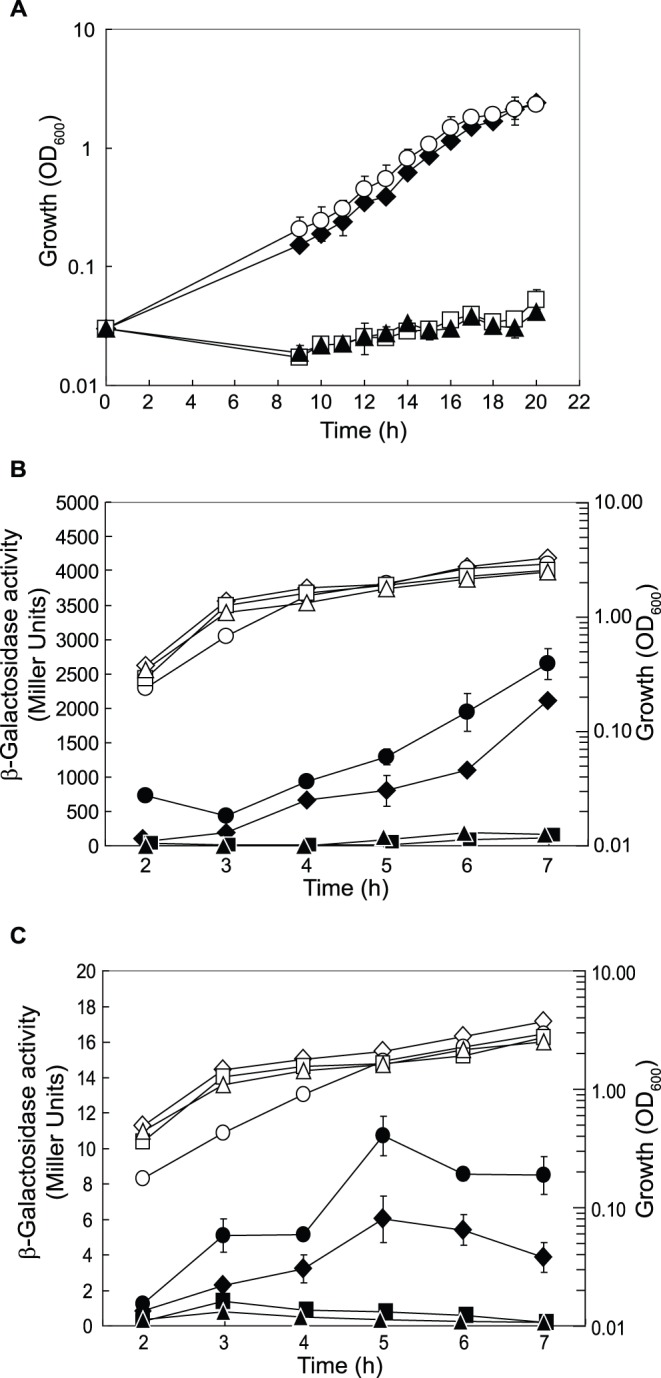
Utilization of branched-chain amino acids is under CbrAB/Crc control. (**A**) Growth of PAO1 (black diamonds), PAO1Δ*cbrB* (black triangles), PAO1Δ*crcZ* (white squares) and PAO1Δ*crc* (white circles) was monitored in BSM supplemented with 23 mM leucine, 25.6 mM valine and 7.6 mM isoleucine as the sole carbon sources. β-Galactosidase activities conferred by (**B**) a translational *bkdA1’-‘lacZ* fusion (pME10049) and (**C**) a translational *bkdR’-‘lacZ* fusion (pME10048) were measured in PAO1 (black diamonds), PAO1Δ*cbrB* (black triangles), PAO1Δ*crcZ* (black squares) and PAO1Δ*crc* (black circles). Cells were grown in LB supplemented with 23 mM leucine, 25.6 mM valine and 7.6 mM isoleucine. The corresponding growth curves are shown in white symbols.

*Targets of the CbrAB/Crc system in the uptake and metabolism of amino acids -* The CbrAB two-component system is important for maintaining the C to N balance during amino acid utilization by *P. aeruginosa* [23]. In a seminal study, Crc was found to be a post-transcriptional regulator of the *bkd* operon, which is involved in the utilization of branched-chain amino acids in *P. putida* and *P. aeruginosa* [24]. Furthermore, a recent proteomic analysis [7] revealed that Crc is involved in the regulation of several transporters required for the uptake of amino acids in *P. aeruginosa.* Our transcriptome analysis revealed a number of CbrAB/Crc-regulated genes and operons (e.g., *bkdA1A2B-lpdV*, *braBCDEDG*, *aroP2*, *aruBEFG*, *aotP*, *arcBC*) that are involved in the utilization and transport of amino acids. In agreement with the original observations of Hester *et al*. [24], we found that the CbrAB/Crc system is involved in the regulation of branched-chain amino acid utilization in *P. aeruginosa*. In BSM supplemented with valine, isoleucine and leucine as the sole carbon sources, strains PAO1Δ*crcZ* and PAO1Δ*cbrB* were not able to grow, whereas strains PAO1 and PAO1Δ*crc* did grow (Figure 4A). Branched-chain amino acid assimilation requires an initial deamination step performed by the branched-chain amino acid transaminase (encoded by *ilvE*), followed by decarboxylation by the branched-chain keto acid dehydrogenase (encoded by the *bkdA1A2B*-*lpdV* operon). The *bkd* operon is induced by branched-chain amino acids as well as branched-chain keto acids [25]. In *P. putida,* all five genes are repressed by Crc [26], whereas in *P. aeruginosa* only the *bkd* operon, but not the *ilvE* gene, seems to be under catabolite repression control by Crc (Tables 1 and 2). To verify the results from our transcriptome data, we analyzed the expression of a translational *bkdA1’*-*‘lacZ* fusion in LB supplemented with 23 mM leucine, 25.6 mM valine and 7.6 mM isoleucine. As shown in Figure 4B, *bkdA1’-‘lacZ* expression was slightly increased in PAO1Δ*crc* by comparison with that in PAO1, and strongly repressed in PAO1Δ*crcZ* and PAO1Δ*cbrB*. In *P. putida*, a CA motif (AAAAACAA) is located 24 nucleotides upstream of the *bkdA1* start codon. By contrast, in *P. aeruginosa* a putative CA motif (AAAAACAAAA) occurs 187 nucleotides upstream of the *bkdA1* start codon and it is questionable whether this sequence would affect translation initiation of *bkdA1*. A more likely scenario pictures the *bkdR* gene, which lies upstream of the *bkd* operon, as a target of the CbrAB/Crc system. In *P. putida*, the transcriptional regulator BkdR positively controls expression of the *bkd* operon [27]. The BkdR protein content was elevated in a *crc* mutant while the *bkdR* mRNA levels were unchanged, whereas *bkdA1* expression was affected at both the mRNA and the protein level [28].

In *P. aeruginosa*, two putative CA motifs can be found in the untranslated leader region of *bkdR* (AACUACAAGAA at −36 to −26 and AACAAGAGAAACAA at −20 to −7). To see whether the CbrAB/Crc system regulates the expression of *bkdR*, we performed β-galactosidase assays of a translational *bkdR’-‘lacZ* fusion in LB supplemented with 23 mM leucine, 25.6 mM valine and 7.6 mM isoleucine. The activity of this fusion was 1.5- to 2.0-fold increased in PAO1Δ*crc* compared to PAO1 and was practically undetectable in PAO1Δ*crcZ* and PAO1Δ*cbrB* (Figure 4C), confirming that *bkdR* is a target of the CbrAB/Crc system in *P. aeruginosa*.

**Figure 5:**
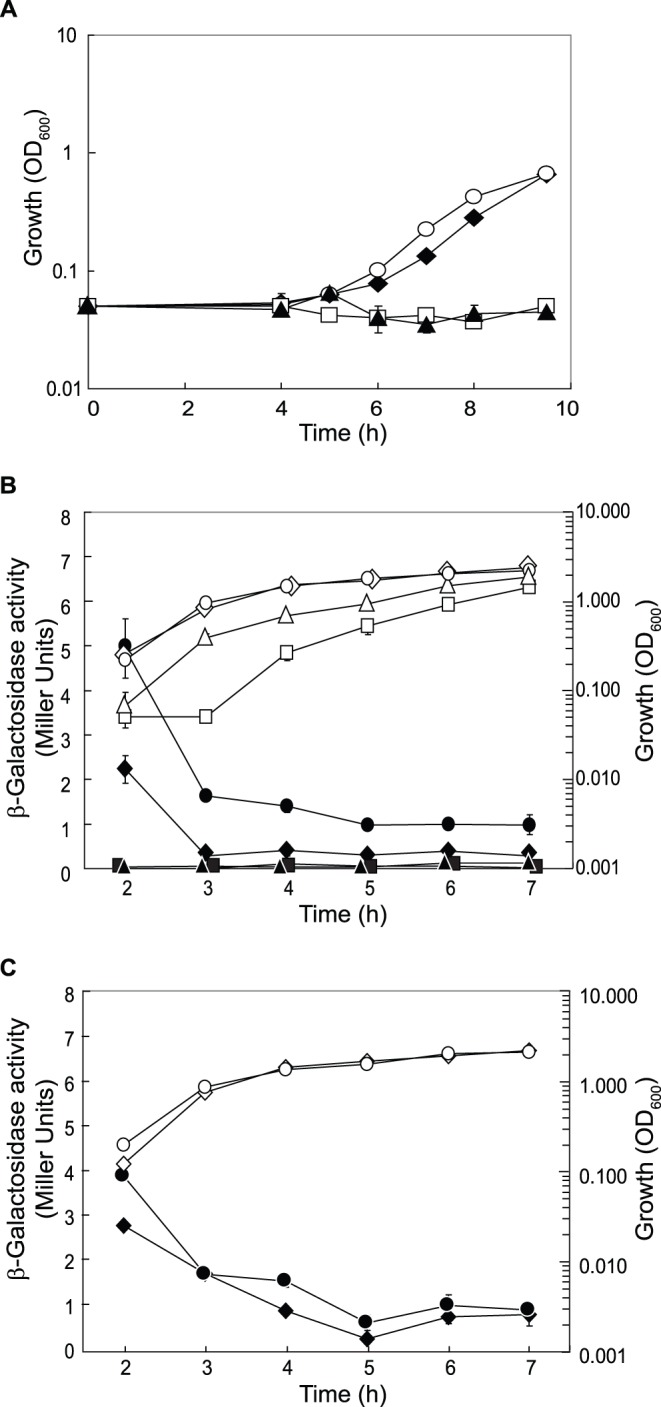
Tyrosine uptake is under CbrAB/Crc control. (**A**) Growth of PAO1 (black diamonds), PAO1Δ*cbrB* (black triangles), PAO1Δ*crcZ* (white squares) and PAO1Δ*crc* (white circles) was followed in BSM supplemented with 4 mM tyrosine as the sole carbon source. (**B**) β-Galactosidase activities conferred by the translational *aroP2’-‘lacZ* fusion (pME10046) were determined in PAO1 (black diamonds), PAO1Δ*cbrB* (black triangles), PAO1Δ*crcZ* (black squares) and PAO1Δ*crc* (black circles). (**C**) Expression of a translational *aroP2’-‘lacZ* fusion with a mutated CA motif (pME10047) was similarly followed in PAO1 (black diamonds) and PAO1Δ*crc* (black circles). Cells were grown in synthetic sputum medium (SCFM). The corresponding growth curves are shown in white symbols.

Another good candidate for interaction with the CbrAB/Crc system was the *aroP2* gene, which encodes a transporter for aromatic amino acids. A growth experiment was performed with PAO1 and its *crc*, *crcZ*, and *cbrB* deletion mutants in BSM supplemented with 4 mM tyrosine as the sole carbon source. The *crcZ* and *cbrB* mutants did not grow, whereas PAO1 and PAO1Δ*crc* reached an OD_600_ of 1 after 9 h; PAO1Δ*crc* even grew slightly faster than the wild type strain (Figure 5A). The expression of a translational *aroP2’-‘lacZ* fusion was very low in LB and in BSM amended with tyrosine (data not shown), as would be expected for a gene specifying an inner membrane protein. It has been shown previously that *aroP2* expression is up-regulated in a lyophilized CF sputum medium, which is rich in amino acids [29]. Therefore, we used a synthetic CF sputum medium (SCFM; [30]) for measuring the β-galactosidase activities conferred by the *aroP2’-‘lacZ* fusion. Although expression was still very low under these conditions, a 2- to 4-fold higher expression of the *aroP2’-‘lacZ* fusion was measured in PAO1Δ*crc*, compared with PAO1; in the *crcZ* and *cbrB* mutants *aroP2* was fully repressed (Figure 5B). The deletion of the CA motif in the fusion construct resulted in loss of regulation by Crc (Figure 5C) and confirmed that *aroP2* is a target of the CbrAB/Crc system.

**Figure 6:**
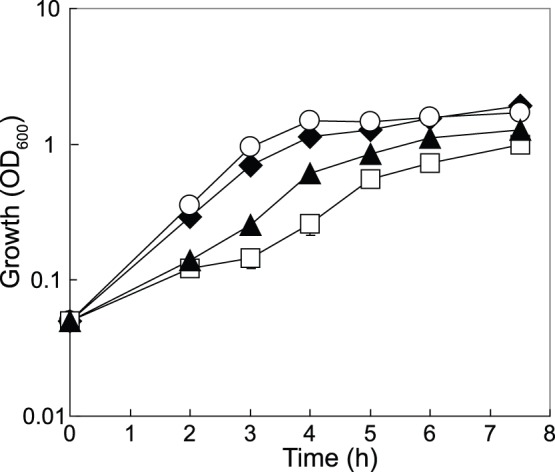
Growth of *P. aerug*inosa in sputum medium is under CbrAB/Crc control. Growth curves of PAO1 (black diamonds), PAO1Δ*cbrB* (black triangles), PAO1Δ*crcZ* (white squares) and PAO1Δ*crc* (white circles) were obtained in SCFM.

*Consequences of catabolite repression for environmental adaptation of* P. aeruginosa - As we have shown that the CbrAB/Crc system strongly affects the utilization and transport of various less preferred carbon and nitrogen sources, the question arises as to what benefits *P. aeruginosa* may obtain from this regulation in natural environments, e.g. in the CF lung where amino acids are important C sources [29], [30]. To investigate the impact of catabolite repression under such conditions, we cultivated PAO1, PAO1Δ*crc*, PAO1Δ*crcZ* and PAO1Δ*cbrB* aerobically in the artificial sputum medium (SCFM). The *crcZ* and *cbrB* deletion mutants grew more slowly than the PAO1 wild type, whereas the *crc* deletion strain grew similarly to PAO1 (Figure 6). In this sputum medium, tyrosine and phenylalanine are important C sources and degradation of both amino acids is induced in *P. aeruginosa* [31]. Moreover, leucine (1.6 mM), isoleucine (1.1 mM) and valine (1.1 mM) are present at high concentrations in this medium [30]. As we have shown above, tyrosine uptake (Figure 4) and utilization of branched-chain amino acids (Figure 5) are tightly controlled by the CbrAB/Crc system. The growth patterns seen in SCFM (Figure 6) are fully consistent with this regulation. As during chronic infection *P. aeruginosa* forms biofilms [32], we also measured biofilm formation in PAO1, PAO1Δ*crc*, PAO1Δ*crcZ* and PAO1Δ*cbrB* in SCFM using a static biofilm assay. The *crcZ* and *cbrB* mutants were impaired in biofilm formation whereas the biofilm formed by PAO1Δ*crc* was increased ∼5-fold, by comparison with the wild type (Figure S2). In BSM-succinate medium we observed the same pattern as that already seen in SCFM (Figure S2). However, when we used BSM medium supplemented with an intermediate (glucose) or non-preferred carbon source (mannitol), the repressing effect of Crc on biofilm formation was lost (Figure S2). In conclusion, global regulation by the CbrAB/Crc system may give the wild type *P. aeruginosa* a selective advantage over *crcZ* or *cbrB* mutants in the CF lung and may help the bacterium to persist in CF patients.

## Discussion

In our present transcriptome analysis, the expression of ≥380 genes was affected by mutations in the CbrAB/Crc system in *P. aeruginosa* when we take into account data obtained in LB and in the defined medium BSM-succinate. Thus, in spite of a major output at the level of translation, the CbrAB/Crc signal transduction pathway clearly has a global impact on transcript abundance. In agreement with earlier phenotypic data [1], the present transcriptomic data indicate that most CbrB activity is linked to CrcZ activity (Figure 1) in that transcriptomic responses were similar, but not identical in *cbrB* and *crcZ* mutants (Tables S1 and S2). It is therefore possible that CbrB could act as a transcriptional regulator of genes other than the previously recognized *crcZ*, *lipA* and *hutU* genes [5]. However, such novel CbrB target genes remain to be identified. Whether CrcZ regulates mRNA expression independently of Crc is another question that cannot be answered by the experiments of this study.

The overlap between Crc- and CrcZ-regulated transcripts was unexpectedly low (Figure 1). There may be several reasons for this finding. In general, mRNAs are more stable when they are translated [33]. Nevertheless, in a *crc* mutant, derepressed translation may not always result in more stable, hence more abundant transcripts, whereas in a *crcZ* mutant, in which translational initiation of target mRNAs is repressed, the (negative) effects on transcript abundance may be more obvious. However, under both growth conditions used here, expression of *crcZ* is low and thus Crc-mediated repression of target mRNAs by Crc is strong [1]. Therefore, a *crcZ* mutation may result only in minor and possibly undetectable changes of the expression of some genes. As shown in Table 2, RT-qPCR (as a more sensitive technique than the microarray analysis) established that the *estA*, *acsA*, *bkdA1* and *aroP2* transcripts were regulated by Crc and CrcZ/CbrB in an opposite manner. However, there are also limitations. For example, *bkdR* did not reveal changes of transcript levels, whereas a *bkdR’-‘lacZ* reporter fusion was regulated (Table 2). Finally, many genes were not induced at the transcriptional level under the growth conditions used, making changes due to catabolite repression difficult to detect. Despite such limitations, we have been able to use transcriptome analysis as a tool to identify several novel targets of the CbrAB/Crc system.

The main targets of the CbrAB/Crc system seem to be metabolic transcripts, which is expected for a catabolite repression control system. Moreover, as noted before [6], [7], [34], the CbrAB/Crc system not only regulates genes for the utilization of less preferred C sources, but also dramatically influences motility and biofilm development, traits that are involved in the pathogenicity of *P. aeruginosa* and that depend on nutritional conditions. For example, a defect in CbrAB-mediated regulation of aromatic and branched-chain amino acid utilization affected growth and biofilm development of *P. aeruginosa* in sputum medium (Figures 6 and S2). In addition to *aroP2* and *bkdR* (Figures 4 and 5), the *braC* gene and the *braDEFG* operon (encoding transport proteins with high affinity for the branched-chain amino acid leucine, isoleucine and valine and with low affinity for alanine and threonine; [35], [36]), were down-regulated in the *crcZ* mutant in both media and up-regulated in the *crc* mutant in BSM (Table 1). An incomplete CA motif (UACAACAA at +23 to +30) in the coding region of *braD* as well as an extended, but imperfect CA motif (ACAACAAUGACAACA at −13 to −6) upstream of the *braC* start codon suggests that the CbrAB/Crc system also controls these genes. However, further investigation is required to confirm this regulation.

In sputum medium a *crc* mutant had no growth advantage over the wild type (Figure 6) and there is currently no evidence that colonization of the CF lung by *P. aeruginosa* would select for *crc* mutants. However, both the *crcZ* and the *cbrB* mutant were partially deficient for growth in SCFM and may therefore be affected in virulence.

It has been known for some time that nutritional conditions influence biofilm development [37], [38] and that the CbrAB/Crc system is an important regulator for biofilm formation, although some published results appear to be strain-specific [6], [7], [34], [39]. For example, a PA14 Δ*crc* mutant produced less biofilm in minimal media containing glucose [6], [34] whereas a PAO1 Δ*crc* strain showed enhanced biofilm formation in LB compared with the wild type strain [7]. Our data indicate that the impact of the CbrAB/Crc system on biofilm development depends on media and carbon sources (Figure S2). In BSM medium, different carbon sources clearly affected the formation of static biofilms (Figure S2), which is consistent with different carbon sources having different regulatory effects on targets of the CbrAB/Crc system. By contrast, in LB medium the typical pattern of upregulation in the *crc* mutant and strong repression in the *crcZ* and *cbrB* deletion strains was not observed. In conclusion, the CbrAB/Crc system acts on a variety of different transcripts involved in biofilm development. Finding out more about the relationships between nutrient availability, catabolite repression control and regulation of virulence and biofilm development in *P. aeruginosa* may ultimately lead to a better understanding of the bacterium’s pathogenicity and intrinsic resistance to antimicrobial agents.

## Methods

*Bacterial strains and growth conditions -* The strains and plasmids used in this study are listed in Table S3. Unless indicated otherwise, cells were grown in Luria Broth (LB) [12], [40] or in a minimal medium (BSM) [41] supplemented with 40 mM succinate [1]. For the investigation of special targets and growth behavior we used BSM amended with either 4 mM tyrosine, 5 mM succinate +40 mM acetate, 40 mM acetate or 23 mM leucine +25.6 mM valine +7.6 mM isoleucine as the sole carbon sources and LB supplemented with 23 mM leucine +25.6 mM valine +7.6 mM isoleucine. The synthetic sputum medium SCFM has been described by Palmer *et al*. [30]. When required, antibiotics were added to the media at the following concentrations: 100 µg ml^−1^ ampicillin for *Escherichia coli* and 125 µg ml^−1^ tetracycline for *P. aeruginosa*.

*Transcriptome analysis -* Overnight cultures of *P. aeruginosa* PAO1, the Δ*cbrB* mutant PAO6711, the Δ*crc* mutant PAO6673 and the Δ*crcZ* mutant PAO6679 were diluted to an initial OD_600_ of 0.05 in 20 ml of LB or BSM amended with 40 mM succinate. The cultures were grown at 37°C with vigorous shaking until they reached an OD_600_ of 1.6. RNA purification, cDNA synthesis and cDNA hybridization were performed as described previously [42]. Processing of the *P. aeruginosa* GeneChip Array (Affymetrix) was performed at the University of Lausanne Center for Integrative Genomics. For each condition, cultures were grown in triplicate, and RNAs from these cultures were pooled before proceeding to cDNA synthesis. In addition, one biological replicate for each condition was performed on a separate day and run on separate microarray chips. Transcripts tabulated in Tables S1 and S2 meet the following criteria: (i) the *P* value for each transcript analyzed is ≤0.05 and (ii) the change in transcript level is ≥2.0-fold. In compliance to MIAME guidelines. The data has been deposited in the GEO database, GEO accession number: GSE33245.

*Real-time quantitative PCR (RT-qPCR) –* PAO1, PAO1Δ*cbrB* mutant PAO6711, PAO1Δ*crc* mutant PAO6673 and PAO1Δ*crcZ* mutant PAO6679 were grown in LB medium or BSM supplemented with 40 mM succinate at 37°C with vigorous shaking until they reached an OD_600_ of 1.6. Cells were harvested using the RNA bacteria protect solution (QIAGEN). Total RNA was extracted with the RNA RNeasy kit (QIAGEN), treated with RQ1 DNase (Promega) to remove contaminating genomic DNA and subsequently re-purified using phenol-chlorophorm extraction. cDNA from each sample was obtained as previously described [16] for every sample 500 ng of total RNA was used. The resulting cDNAs were used as templates for qPCR and quantitated with a Bio-Rad iCycler machine using a Sybr Green Quantitect kit (QIAGEN). Fold changes were estimated by a comparative threshold cycle method with the *anr* gene PA1544 as a standard [43]. The primer pairs, termed gene name fw and gene name rev, used for qRT-PCR are shown in Table S4.

*Construction of plasmids -* To construct translational *‘lacZ* fusions to *estA*, *bkdA* and *bkdR*, fragments of 592 bp, 354 bp and 368 bp, respectively, were amplified by PCR using the primer pairs Q67_estAfw/R67_estArev (*estA*), PA2246fw/PA2246rev (*bkdA*) and PA2247fw/PA2247rev (*bkdR*) (Table S4) and chromosomal DNA of strain PAO1 as template. The fragments containing the respective authentic promoter regions and the translation initiation sites were fused in-frame after the 6^th^ codon of *estA* and the 4^th^ codon of the *bkd*A and *bkdR* genes to the 8^th^ codon of *lacZ*. These fragments were digested with *Eco*RI and *Bam*HI and cloned into the corresponding sites of pME6015 generating pTLestA (*estA’-‘lacZ*), pME10049 (*bkdA1’-‘lacZ*) and pME10048 (*bkdR’-‘lacZ*). The translational *acsA*’-‘*lacZ* and *aroP2*’-‘*lacZ* fusions were constructed as described above by amplifying a 202-bp and 316-bp PCR fragment with primers AcsA1/AcsA2 and AroP21/AroP22 (Table S4), respectively. These fragments were digested with *Eco*RI and *Pst*I and cloned into the corresponding sites of pME6015, resulting in plasmid pME10044 (*acsA*’-‘*lacZ*) and pME10046 (*aroP2*’-‘*lacZ*).

The CA motif mutations (TCAGTAGC instead of AAAAACAA) were introduced into pTL*estA* according to the QuikChange^(R)^ site-directed mutagenesis protocol (http://www.stratagene.com/manuals/200518.pdf) with the mutagenesis primers L71_estAmut1a and M71_estAmut1b (Table S4). The parental DNA template was digested with *Dpn*I, and the mutated plasmid was transformed into *E. coli* XL1-Blue, generating pTL*estA*-ΔCA. The same procedure was used to mutate the CA motif sequences AACAAAAACAA of *acsA* and AACAATAA of *aroP2* with the mutagenesis primer pairs AcsAmutfw/AcsAmutrev and AroP2mutfw/AroP2mutrev (Table S4), respectively. The resulting plasmids were named pME10045 (*acsA*-ΔCA) and pME10047 (*aroP2*-ΔCA). All mutations were confirmed by sequencing.

*β-Galactosidase assays -* β-Galactosidase activities were quantified by the Miller method [40], using cells permeabilized with 5% toluene or 5% (v/v) chloroform and 0.01% SDS. Values shown were derived from three independent experiments and are means ± standard deviation.

*Static biofilm assay -* A static-culture biofilm assay in microtiter plates was performed as described previously [44]. Cells were grown in LB, SCFM or BMS medium supplemented with 40 mM succinate, glucose or mannitol, respectively, for 24 h and biofilms were stained with 0.1% crystal violet solution. The dye bound, which is proportional to the biofilm produced, was solubilized with 96% (v/v) ethanol and the absorption was photometrically measured at 540 nm (A_540_).

## Supporting Information

Figure S1 β**-Galactosidase activity measurements of** ***amiE’-‘lacZ*** **(pME9655) in PAO1 (black diamonds) and PAO1**Δ***crc*** **(white circles) performed in LB.**
(EPS)

Figure S2 **Biofilm formation of PAO1 and** Δ***crc*** **,** Δ***crcZ*** **,** Δ***cbrB*** **mutant strains.** Static biofilm development of PAO1 (black bar), PAO1Δ*crc* (white bar), PAO1Δ*crcZ* (grey bars) and PAO1Δ*cbrB* (dashed bar) was measured after growth for 24 h in SCFM or BSM supplemented with 40 mM succinate (Succinate) or 40 mM glucose (Glucose) or 40 mM mannitol (Mannitol) in a polyethylene microtiter plate. The biofilm was stained with crystal violet and after stripping with 96% (v/v) ethanol the A_540_ was photometrically determined.
(EPS)

Table S1 **Transcripts which were at least two-fold differentially expressed in PAO1Δ** ***crc*** **compared to PAO1.**
(DOC)

Table S2 **Transcripts which were differentially regulated in the** ***cbrB*** **and** ***crcZ*** **mutants compared to PAO1.**
(DOC)

Table S3 **Strains and plasmids used in this study.**
(DOC)

Table S4 **DNA oligonucleotides used in this study.**
(DOC)
