# Supplementary material for: Novel Targets of the CbrAB/Crc Carbon Catabolite Control System Revealed by Transcript Abundance in Pseudomonas aeruginosa
Source: PLoS One. 2012 Oct 24;7(10):e44637. doi: 10.1371/journal.pone.0044637 (PMC3480352; doi:10.1371/journal.pone.0044637)
Supplement: Table S1 — Transcripts which were at least two-fold differentially expressed in PAO1Δ crc compared to PAO1. (DOC) [file pone.0044637.s003.doc]

**Table S1.** Transcripts which were at least two fold differentially expressed in PAO1Δ*crc* compared to PAO1.

| **ORF** | **Gene** | **LB** | **BSM +succinate** | **Description** | **CA motif (location)** |
| --- | --- | --- | --- | --- | --- |
| PA0044 | *exoT* |  | -7.07 | exoenzyme T |  |
| PA0493 |  | -4.78 |  | probable biotin-requiring enzyme |  |
| PA0494 |  | -3.43 |  | probable acyl-CoA carboxylase subunit |  |
| PA0510 | *nirE* | -2.64 |  | probable uroporphyrin-III c-methyltransferase |  |
| PA0511 | *nirJ* | -4.50 |  | heme d1 biosynthesis protein |  |
| PA0514 | *nirL* | -4.09 |  | heme d1 biosynthesis protein |  |
| PA0515 | *nirD* | -5.26 |  | probable transcriptional regulator |  |
| PA0516 | *nirF* | -4.95 |  | heme d1 biosynthesis protein |  |
| PA0517 | *nirC* | -4.75 |  | probable c-type cytochrome precursor |  |
| PA0518 | *nirM* | -7.88 |  | cytochrome c-551 precursor |  |
| PA0519 | *nirS* | -5.72 |  | nitrite reductase precursor |  |
| PA0523 | *norC* | -13.93 |  | nitric-oxide reductase subunit C |  |
| PA0524 | *norB* | -10.24 |  | nitric-oxide reductase subunit B |  |
| PA0572 |  |  | -3.77 | hypothetical protein |  |
| PA0752 |  | 3.32 |  | hypothetical protein |  |
| PA0753 |  | 2.91 |  | hypothetical protein |  |
| PA0754 |  | 3.98 |  | hypothetical protein | GAACAAGAA (-24 to -17) |
| PA0755 | *opdH* | 5.90 | 2.05 | *cis*-aconitate porin OpdH | AAGAACAA (-25 to -18) |
| PA0807 | *ampDh3* | | -2.07 | AmpDh3 |  |
| PA0866 | *aroP2* | 4.73 |  | aromatic amino acid transport protein | AACAAUAA (-33 to -12) |
| PA0887 | *acsA* | -3.41 | 7.98 | acetyl-coenzyme A synthetase | AACAAAAACAA (-35 to -25) |
| PA0996 | *pqsA* |  | -2.01 | probable coenzyme A ligase |  |
| PA0997 | *pqsB* |  | -2.11 | PqsB protein |  |
| PA0998 | *pqsC* |  | -2.01 | PqsC protein |  |
| PA0999 | *pqsD* |  | -2.00 | 3-oxoacyl-[acyl-carrier-protein] synthase III |  |
| PA1000 | *pqsE* |  | -2.09 | quinolone signal response protein |  |
| PA1001 | *phnA* |  | -2.08 | anthranilate synthase component I |  |
| PA1002 | *phnB* |  | -2.01 | anthranilate synthase component II |  |
| PA1071 | *braF* |  | 2.08 | branched-chain amino acid transport protein BraF |  |
| PA1072 | *braE* |  | 2.33 | branched-chain amino acid transport protein BraE |  |
| PA1073 | *braD* |  | 2.50 | branched-chain amino acid transport protein BraD |  |
| PA1074 | *braC* |  | 2.14 | branched-chain amino acid transport protein BraC |  |
| PA1123 |  | -6.00 |  | hypothetical protein |  |
| PA1183 | *dctA* | -4.30 |  | C4-dicarboxylate transport protein |  |
| PA1228 |  | 3.33 |  | hypothetical protein |  |
| PA1246 | *aprD* |  | -2.00 | alkaline protease secretion protein AprD |  |
| PA1337 | *ansB* | -3.65 |  | glutaminase-asparaginase |  |
| PA1339 |  | -2.26 |  | probable ATP-binding component of ABC transporter |  |
| PA1342 |  | -2.68 |  | probable binding protein component of ABC transporter | AAUAAAAA (-33 to -26) |
| PA1556 | *ccoO2* | -3.30 |  | cytochrome c oxidase. cbb3-type. CcoO subunit |  |
| PA1561 | *aer* | -2.44 |  | aerotaxis receptor |  |
| PA1579 |  |  | -2.02 | hypothetical protein |  |
| PA1617 |  |  | 2.44 | probable AMP-binding enzyme | AACAACAACAA (-23 to -13) |
| PA1633 | *kpdA* | 2.70 |  | potassium transporting ATPase |  |
| PA1634 | *kpdB* | 2.40 |  | potassium transporting ATPase |  |
| PA1650 |  |  | 2.19 | probable transporter | AAUAACAAUAA (-10 to -1) |
| PA1657 |  |  | -2.44 | conserved hypothetical protein |  |
| PA1658 |  |  | -2.29 | conserved hypothetical protein |  |
| PA1659 |  |  | -2.47 | hypothetical protein |  |
| PA1694 | *pscQ* |  | -3.39 | translocation protein in type III secretion |  |
| PA1706 | *pcrV* |  | -4.45 | type III secretion protein PcrV |  |
| PA1707 | *pcrH* |  | -7.83 | regulatory protein PcrH |  |
| PA1708 | *popB* |  | -10.61 | translocator protein PopB |  |
| PA1709 | *popD* |  | -9.41 | translocator protein PopD |  |
| PA1710 | *exsC* |  | -2.56 | exoenzyme S synthesis protein C precursor |  |
| PA1711 | *exsE* |  | -2.62 | ExsE |  |
| PA1712 | *exsB* |  | -2.64 | exoenzyme S synthesis protein B |  |
| PA1714 | *exsD* |  | -2.24 | ExsD |  |
| PA1718 | *pscE* |  | -4.49 | type III export protein PscE |  |
| PA1719 | *pscF* |  | -3.58 | type III export protein PscF |  |
| PA1720 | *pscG* |  | -2.38 | type III export protein PscG |  |
| PA1721 | *pscH* |  | -2.01 | type III export protein PscH |  |
| PA1722 | *pscI* |  | -2.72 | type III export protein PscI |  |
| PA1763 |  |  | 2.10 | hypothetical protein | AAAAACAAGAACAA (-31 to -18) |
| PA1892 |  |  | 4.19 | hypothetical protein |  |
| PA1893 |  |  | 2.83 | hypothetical protein |  |
| PA1894 |  |  | 5.83 | hypothetical protein |  |
| PA1895 |  |  | 4.27 | hypothetical protein |  |
| PA1896 |  |  | 7.56 | hypothetical protein |  |
| PA1897 |  |  | 7.15 | hypothetical protein |  |
| PA1984 | *exaC* |  | 3.84 | NAD+ dependent aldehyde dehydrogenase ExaC |  |
| PA1992 | *ercS* |  | 2.16 | two-component sensor ErcS |  |
| PA2001 | *atoB* | 2.40 |  | acetyl-CoA acetyltransferase |  |
| PA2113 | *opdO* | 4.47 |  | pyroglutamate porin OpdO |  |
| PA2191 | *exoY* |  | -2.16 | adenylate cyclase ExoY |  |
| PA2247 | *bkdA1* |  | 3.23 | 2-oxoisovalerate dehydrogenase (alpha subunit) |  |
| PA2321 |  | 2.60 |  | gluconokinase |  |
| PA2379 |  | 2.45 |  | probable oxidoreductase |  |
| PA2453 |  |  | -3.05 | hypothetical protein |  |
| PA2533 |  |  | 2.13 | probable sodium:alanine symporter | AACAAGAAUAA (-20 to -10) |
| PA2566 |  |  | -2.20 | conserved hypothetical protein |  |
| PA2780 |  |  | -2.08 | hypothetical protein |  |
| PA3038 |  |  | 16.75 | probable porin | AAUAACAA (-7 to +1) |
| PA3079 |  |  | 2.83 | hypothetical protein | AACAACAACAAUAA  (-26 to -13) |
| PA3187 |  | -7.08 |  | probable ATP-binding component of ABC transporter |  |
| PA3188 |  | -4.74 |  | probable permease of ABC sugar transporter |  |
| PA3189 |  | -2.49 |  | probable permease of ABC sugar transporter |  |
| PA3190 |  | -7.43 |  | probable binding protein component of ABC sugar transporter | AAUAACAA (-24 to -17) |
| PA3234 |  |  | 4.00 | probable sodium:solute symporter |  |
| PA3235 |  |  | 7.16 | conserved hypothetical protein | AAAAACAA (-7 to +1) |
| PA3361 | *lecB* |  | -5.22 | fucose-binding lectin PA-IIL |  |
| PA3363 | *amiR* | 2.74 |  | aliphatic amidase regulator |  |
| PA3365 | *amiB* | 3.66 |  | probable chaperone |  |
| PA3366 | *amiE* | 4.65 |  | aliphatic amidase | AACAACAA (-20 to -13) |
| PA3392 | *nosZ* | -8.77 |  | nitrous-oxide reductase precursor |  |
| PA3452 | *mqoA* | 4.87 |  | malate:quinone oxidoreductase |  |
| PA3478 | *rhlB* |  | -7.33 | rhamnosyltransferase chain B |  |
| PA3535 |  |  | 2.66 | probable serine protease |  |
| PA3570 | *mmsA* |  | 2.23 | methylmalonate-semialdehyde dehydrogenase | AACAAUAA (-37 to -30) |
| PA3584 | *glpD* | -6.00 |  | glycerol-3-phosphate dehydrogenase |  |
| PA3841 | *exoS* |  | -6.55 | exoenzyme S |  |
| PA3842 | *spcS* |  | -5.40 | specific Pseudomonas chaperone for ExoS. SpcS |  |
| PA3843 |  |  | -2.99 | hypothetical protein |  |
| PA3866 |  |  | -2.58 | pyocin protein |  |
| PA3872 | *narI* | -5.48 |  | respiratory nitrate reductase gamma chain |  |
| PA3873 | *narJ* | -4.64 |  | respiratory nitrate reductase delta chain |  |
| PA3874 | *narH* | -7.90 |  | respiratory nitrate reductase beta chain |  |
| PA3875 | *narG* | -6.01 |  | respiratory nitrate reductase alpha chain | AAGAAGAA (+34 to +41) |
| PA3876 | *narK2* | -3.09 |  | nitrite extrusion protein 2 |  |
| PA3877 | *narK1* | -4.08 |  | nitrite extrusion protein 1 |  |
| PA3904 |  | -2.44 |  | hypothetical protein |  |
| PA3915 | *moaB1* | -4.89 |  | molybdopterin biosynthetic protein B1 |  |
| PA4134 |  |  | -2.21 | hypothetical protein |  |
| PA4139 |  |  | -3.37 | hypothetical protein |  |
| PA4147 | *acoR* |  | 3.38 | transcriptional regulator AcoR | AACAACAA (-30 to -23) |
| PA4150 |  |  | 2.77 | probable dehydrogenase E1 component | AACAACAA (-9 to -2) |
| PA4151 | *acoB* |  | 4.10 | acetoin catabolism protein AcoB | AACAAGAA (-22 to -15) |
| PA4152 |  |  | 3.69 | probable hydrolase |  |
| PA4153 |  |  | 3.96 | 2.3-butanediol dehydrogenase |  |
| PA4198 |  |  | 2.42 | probable AMP-binding enzyme |  |
| PA4306 | *flp* |  | -2.90 | Type IVb pilin. Flp | AACAAGAA (-22 to -15) |
| PA4496 |  |  | 2.90 | probable binding protein component of ABC transporter |  |
| PA4500 |  |  | 2.03 | probable binding protein component of ABC transporter | AAAAAGAAAAAA  (-22 to -11) |
| PA4501 | *opdD* |  | 2.64 | Glycine-glutamate dipeptide porin OpdP | AACAAUAA (-37 to -30) |
| PA4587 | *ccpR* | -6.19 |  | cytochrome c551 peroxidase precursor |  |
| PA4619 |  | 2.13 |  | probable c-type hypothetical protein cytochrome |  |
| PA4770 | *lldP* |  | 2.96 | L-lactate permease | AACAACAA (-25 to -18) |
| PA4909 |  |  | 5.50 | probable ATP-binding component of ABC transporter |  |
| PA4910 |  |  | 6.60 | probable ATP-binding component of ABC transporter |  |
| PA4911 |  |  | 2.36 | probable permease of ABC branched-chain amino acid transporter |  |
| PA4912 |  |  | 7.43 | probable permease of ABC branched chain amino acid transporter |  |
| PA4913 |  |  | 6.89 | probable binding protein component of ABC transporter | AACAACAA (-53 to -46) |
| PA4920 | *nadE* | -2.17 |  | NH3-dependent NAD synthetase |  |
| PA5112 | *estA* |  | 2.00 | esterase EstA | AAAAACAA (-24 to -17) |
| PA5153 |  |  | 2.92 | probable periplasmic binding protein |  |
| PA5154 |  |  | 3.03 | probable permease of ABC transporter |  |
| PA5167 | *dctP* |  | 3.37 | probable C4-dicarboxylate-binding protein | AAGAACAA (-20 to -13) |
| PA5168 | *dctQ* |  | 6.54 | probable dicarboxylate transporter | AAUAAGAA (-20 to -13) |
| PA5169 | *dctM* |  | 6.72 | probable C4-dicarboxylate transporter |  |
| PA5172 | *arcB* | -2.62 |  | catabolic ornithine, carbamoyltransferase |  |
| PA5173 | *arcC* | -3.41 |  | carbamate kinase |  |
| PA5220 |  |  | 2.38 | hypothetical protein | AAGAACAACAAGAA  (-31 to -18) |
| PA5348 |  |  | 5.54 | probable DNA-binding protein | AACAACAA (-26 to -19) |
| PA5446 |  | 3.11 |  | hypothetical protein |  |
| PA5530 |  | 16.12 | 2.58 | probable MFS dicarboxylate transporter |  |
| PA5542 |  |  | 2.74 | hypothetical protein |  |
| PA5543 |  |  | 3.16 | hypothetical protein |  |
| PA5544 |  |  | 2.56 | conserved hypothetical protein |  |
| PA5545 |  |  | 2.87 | conserved hypothetical protein |  |
