# Supplementary material for: Novel Targets of the CbrAB/Crc Carbon Catabolite Control System Revealed by Transcript Abundance in Pseudomonas aeruginosa
Source: PLoS One. 2012 Oct 24;7(10):e44637. doi: 10.1371/journal.pone.0044637 (PMC3480352; doi:10.1371/journal.pone.0044637)
Supplement: Table S2 — Transcripts which were differentially regulated in the cbrB and crcZ mutants compared to PAO1. (DOC) [file pone.0044637.s004.doc]

**Table S2.** Transcripts which were differentially regulated in the *cbrB* and *crcZ* mutants compared to PAO1

|  |  | **LB** | **LB** | **BSM +succinate** | **BSM +succinate** |  |
| --- | --- | --- | --- | --- | --- | --- |
| **ORF** | **Gene** | ***cbrB* vs.*wt*** | ***crcZ* vs.*wt*** | ***cbrB* vs.wt** | ***crcZ* vs.wt** | **Description** |
| PA0044 | *exoT* |  |  |  | -3.04 | exoenzyme T |
| PA0059 | *osmC* | 2.26 |  |  |  | osmotically inducible protein |
| PA0105 | *coxB* | 2.48 | 2.27 |  |  | cytochrome c oxidase. subunit II |
| PA0122 |  | 2.28 | 3.03 |  |  | conserved hypothetical protein |
| PA0130 |  |  | -3.25 |  |  | probable aldehyde dehydrogenase |
| PA0131 |  | -2.13 | -3.42 |  |  | hypothetical protein |
| PA0132 |  | -2.11 | -3.54 |  |  | beta-alanine-pyruvate transaminase |
| PA0169 |  |  |  |  | 2.52 | hypothetical protein |
| PA0170 |  |  |  |  | 2.94 | hypothetical protein |
| PA0171 |  |  |  |  | 2.40 | hypothetical protein |
| PA0175 |  |  |  |  | -2.53 | probable chemotaxis protein methyltransferase |
| PA0176 | *aer2* | 2.74 | 2.33 |  | -3.21 | aerotaxis transducer Aer2 |
| PA0178 |  |  | 2.41 |  |  | probable two-component sensor |
| PA0179 |  | 2.88 | 2.96 |  |  | probable two-component response regulator |
| PA0282 | *cysT* |  |  |  | 2.03 | sulfate transport protein CysT |
| PA0291 | *oprE* | -2.17 | -3.18 |  |  | outer membrane porin |
| PA0295 |  |  |  |  | 2.44 | probable periplasmic polyamine binding protein |
| PA0315 |  | 2.65 | 2.38 |  |  | hypothetical protein |
| PA0328 |  |  | 3.56 |  |  | hypothetical protein |
| PA0355 | *pfpI* | 2.40 |  |  |  | protease PfpI |
| PA0423 | *pasP* |  |  |  | -2.69 | protease PasP |
| PA0434 |  |  |  |  | -2.04 | hypothetical protein |
| PA0447 | *gcdH* | 2.62 | 2.38 |  |  | glutaryl-CoA dehydrogenase |
| PA0459 |  | 2.80 |  |  |  | probable ClpA/B protease ATP binding subunit |
| PA0472 |  |  |  |  | -3.36 | probable sigma-70 factor |
| PA0482 | *glcB* | 2.58 | 2.43 |  |  | malate synthase G |
| PA0484 |  |  |  |  | -2.04 | conserved hypothetical protein |
| PA0492 |  | 2.05 |  |  |  | conserved hypothetical protein |
| PA0494 |  |  | -2.11 |  |  | probable acyl-CoA carboxylase subunit |
| PA0509 | *nirN* | -2.17 |  |  |  | probable c-type cytochrome |
| PA0510 | *nirE* | -2.21 | -2.08 |  |  | probable uroporphyrin-III c-methyltransferase |
| PA0511 | *nirJ* | -2.94 | -2.85 | 2.62 | 3.98 | heme d1 biosynthesis protein |
| PA0512 | *nirH* | -2.02 |  |  |  | conserved hypothetical protein |
| PA0513 |  |  | -2.09 |  | 2.52 | probable transcriptional regulator |
| PA0514 | *nirL* | -2.44 | -2.42 | 2.30 | 3.39 | heme d1 biosynthesis protein |
| PA0515 | *nirD* | -2.46 | -2.35 | 2.37 | 3.87 | probable transcriptional regulator |
| PA0516 | *nirF* | -2.19 | -2.43 | 2.41 | 4.13 | heme d1 biosynthesis protein |
| PA0517 | *nirC* |  | -2.26 | 3.33 | 5.71 | probable c-type cytochrome precursor |
| PA0518 | *nirM* |  | -2.29 | 3.00 | 6.51 | cytochrome c-551 precursor |
| PA0519 | *nirS* |  |  | 3.16 | 5.24 | nitrite reductase precursor |
| PA0523 | *norC* |  | -2.69 |  |  | nitric-oxide reductase subunit C |
| PA0525 | *norD* |  | -2.44 |  |  | probable dinitrification protein |
| PA0572 |  | 3.33 | 2.78 |  |  | hypothetical protein |
| PA0586 |  | 2.75 | 2.06 |  |  | conserved hypothetical protein |
| PA0587 |  | 3.14 |  |  |  | conserved hypothetical protein |
| PA0588 |  | 3.86 | 3.18 |  |  | conserved hypothetical protein |
| PA0622 |  |  |  |  | -2.37 | probable bacteriophage protein |
| PA0656 |  | 2.02 |  |  |  | probable HIT family protein |
| PA0704 |  |  |  |  | -2.21 | probable amidase |
| PA0795 | *prpC* | -2.03 | -2.74 |  |  | citrate synthase 2 |
| PA0807 | *ampDh3* |  |  |  | -2.02 | AmpDh3 |
| PA0852 | *cbpD* |  | 2.2 |  |  | chitin-binding protein CbpD precursor |
| PA0887 | *acsA* | -2.51 | -3.27 | 2.88 | 3.62 | acetyl-coenzyme A synthetase |
| PA0892 | *atoP* |  | 2.49 |  |  | arginine/ornithine transport protein AtoP |
| PA0893 | *argR* |  | 2.52 |  |  | transcriptional regulator ArgR |
| PA0896 | *aruF* |  | 2.28 |  |  | arginine/ornithine succinyltranferase AI subunit AruF |
| PA0897 | *aruG* |  | 2.33 |  |  | arginine/ornithine succinyltranferase AII subunit AruG |
| PA0899 | *aruB* |  | 3.11 |  |  | succinylarginine dihydrolase |
| PA0900 |  |  | 2.20 |  |  | hypothetical protein |
| PA0901 | *aruE* |  | 2.57 |  |  | succinylglutamate desuccinylase AruR |
| PA0918 |  |  |  |  | 4.07 | cytochrome b561 |
| PA0985 | *pyoS5* |  |  |  | -2.10 | pyocin S5 |
| PA0996 | *pqsA* | -3.55 | -3.49 | -18.34 | -16.67 | probable coenzyme A ligase |
| PA0997 | *pqsB* | -3.72 | -3.49 | -33.37 | -56.50 | PqsB protein |
| PA0998 | *pqsC* | -3.73 | -3.89 | -25.21 | -40.64 | PqsC protein |
| PA0999 | *pqsD* | -3.77 | -3.38 | -23.39 | -34.65 | 3-oxoacyl-[acyl-carrier-protein] synthase III |
| PA1000 | *pqsE* | -2.79 | -2.58 | -18.62 | -22.18 | quinolone signal response protein |
| PA1001 | *phnA* | -3.58 | -3.28 | -18.69 | -23.34 | anthranilate synthase component I |
| PA1002 | *phnB* | -2.04 |  | -11.69 | -11.46 | anthranilate synthase component II |
| PA1003 | *pqsR* |  |  |  | -2.48 | transcriptional regulator PqsR (MvfR) |
| PA1041 |  | 4.80 | 3.09 | -2.35 | -5.42 | probable outer membrane protein |
| PA1070 | *braG* | -2.28 | -3.76 |  | -3.22 | branched-chain amino acid transport protein BraG |
| PA1071 | *braF* | -2.37 | -4.02 |  | -2.91 | branched-chain amino acid transport protein BraF |
| PA1072 | *braE* | -2.21 | -3.11 |  | -3.05 | branched-chain amino acid transport protein BraE |
| PA1073 | *braD* | -2.88 |  |  | -5.56 | branched-chain amino acid transport protein BraD |
| PA1074 | *braC* | -2.11 | -3.60 |  | -2.41 | branched-chain amino acid transport protein BraC |
| PA1123 |  | -6.08 | -5.35 |  |  | hypothetical protein |
| PA1168 |  |  |  | 4.18 | 5.33 | hypothetical protein |
| PA1169 |  |  |  | 2.69 | 3.69 | probable lipoxygenase |
| PA1172 | *napC* |  | 2.02 |  |  | cytochrome c-type protein |
| PA1173 | *napB* | 3.08 | 2.30 |  |  | cytochrome c-type protein |
| PA1174 | *napA* | 2.30 | 2.34 |  |  | periplasmic nitrate reductase protein |
| PA1175 | *napD* | 2.61 | 3.19 |  |  | protein of periplasmic nitrate reductase |
| PA1176 | *napF* | 2.28 | 2.40 |  |  | ferredoxin protein |
| PA1183 | *dctA* |  | -2.08 |  |  | C4-dicarboxylate transport protein |
| PA1190 |  | 2.52 |  |  |  | conserved hypothetical protein |
| PA1202 |  | 2.12 | 2.95 |  |  | probable hydrolase |
| PA1203 |  | 2.41 | 2.58 |  |  | hypothetical protein |
| PA1205 |  | 2.05 |  |  |  | conserved hypothetical protein |
| PA1216 |  | 7.77 | 4.21 |  | -2.20 | hypothetical protein |
| PA1217 |  | 3.94 | 2.30 |  |  | probable 2-isopropylmalate synthase |
| PA1245 |  |  | 2.38 |  |  | hypothetical protein |
| PA1246 | *aprD* |  |  |  | -2.03 | alkaline protease secretion protein AprD |
| PA1249 | *aprA* | 6.35 | 6.39 |  |  | alkaline metalloproteinase precursor |
| PA1256 |  |  | 2.23 |  |  | probable ATP-binding component of ABC transporter |
| PA1288 |  |  | -3.02 |  |  | probable outer membrane protein |
| PA1323 |  | 2.49 |  |  |  | hypothetical protein |
| PA1337 | *ansB* | - 2.26 | -3.22 |  |  | glutaminase-asparaginase |
| PA1338 | *ggt* | - 2.65 | -3.52 |  |  | gamma-glutamyltranspeptidase precursor |
| PA1339 |  |  | -2.50 |  |  | probable ATP-binding component of ABC transporter |
| PA1340 |  | -2.00 | -2.75 |  |  | probable permease of ABC transporter |
| PA1341 |  | -2.16 | -3.02 |  |  | probable permease of ABC transporter |
| PA1342 |  |  | -2.38 |  |  | probable binding protein component of ABC transporter |
| PA1344 |  | 2.02 |  |  |  | probable short-chain dehydrogenase |
| PA1354 |  |  |  |  | -2.09 | hypothetical protein |
| PA1363 |  |  |  |  | -2.05 | probable sigma-70 factor |
| PA1546 | *hemN* |  |  |  | 2.59 | oxygen-independent coproporphyrinogen III oxidase |
| PA1554 | *ccoN1* |  |  |  | 2.18 | cytochrome c oxidase. cbb3-type. CcoN subunit |
| PA1555 | *ccoP2* | -4.55 | -3.18 | 2.46 | 3.82 | cytochrome c oxidase. cbb3-type. CcoP subunit |
| PA1556 | *ccoO2* | -4.01 | -2.90 |  |  | cytochrome c oxidase. cbb3-type. CcoO subunit |
| PA1557 | *ccoN2* | -2.56 |  | 2.46 | 3.46 | cytochrome c oxidase. cbb3-type. CcoN subunit |
| PA1579 |  |  |  |  |  | hypothetical protein |
| PA1581 | *sdhC* |  |  |  | 2.88 | succinate dehydrogenase (C subunit) |
| PA1582 | *sdhD* |  |  |  | 2.76 | succinate dehydrogenase (D subunit) |
| PA1596 | *htpG* |  |  |  | 2.08 | heat shock protein HtpG |
| PA1617 |  |  |  |  |  | probable AMP-binding enzyme |
| PA1657 |  | -2.33 |  |  |  | conserved hypothetical protein |
| PA1677 |  | 2.02 |  |  |  | conserved hypothetical protein |
| PA1706 | *pcrV* |  |  |  | -2.50 | type III secretion protein PcrV |
| PA1707 | *pcrH* |  |  |  | -3.10 | regulatory protein PcrH |
| PA1708 | *popB* | 2.11 |  |  | -4.87 | translocator protein PopB |
| PA1709 | *popD* |  |  |  | -4.74 | translocator protein PopD |
| PA1710 | *exsC* |  |  |  | -2.06 | exoenzyme S synthesis protein C precursor |
| PA1711 | *exsE* |  |  |  | -2.18 | ExsE |
| PA1712 | *exsB* |  |  |  | -2.00 | exoenzyme S synthesis protein B |
| PA1714 | *exsD* |  |  |  | -2.05 | ExsD |
| PA1718 | *pscE* |  |  |  | -2.99 | type III export protein PscE |
| PA1719 | *pscF* |  |  |  | -2.30 | type III export protein PscF |
| PA1733 |  |  |  |  | -2.26 | conserved hypothetical protein |
| PA1745 |  | 2.32 |  |  | -2.81 | hypothetical protein |
| PA1761 |  |  |  |  | -2.74 | hypothetical protein |
| PA1837 |  |  |  |  | 2.95 | hypothetical protein |
| PA1838 | *cysI* |  |  |  | 2.45 | sulfite reductase |
| PA1888 |  |  |  | -2.25 | -5.10 | hypothetical protein |
| PA1894 |  |  |  |  | -4.24 | hypothetical protein |
| PA1895 |  |  |  |  | -3.07 | hypothetical protein |
| PA1897 |  |  |  |  |  | hypothetical protein |
| PA1901 | *phzC2* |  |  | -5.71 | -7.72 | phenazine biosynthesis protein PhzC |
| PA1902 | *phzD2* |  |  | -5.62 | -6.95 | phenazine biosynthesis protein PhzD |
| PA1903 | *phzE2* |  |  | -5.96 | -6.35 | phenazine biosynthesis protein PhzE |
| PA1904 | *phzF2* |  |  | -4.91 | -6.05 | probable phenazine biosynthesis protein |
| PA1905 | *phzG2* |  |  | -5.56 | -10.68 | probable pyridoxamine 5'-phosphate oxidase |
| PA1984 | *exaC* | -2.76 |  |  | -3.37 | NAD+ dependent aldehyde dehydrogenase ExaC |
| PA1985 | *pqqA* | 11.34 | 8.74 |  |  | pyrroloquinoline quinone biosynthesis protein A |
| PA1986 | *pqqB* | 2.47 | 2.52 |  |  | pyrroloquinoline quinone biosynthesis protein B |
| PA1987 | *pqqC* | 3.86 | 3.70 |  |  | pyrroloquinoline quinone biosynthesis protein C |
| PA1988 | *pqqD* | 3.70 | 4.02 |  |  | pyrroloquinoline quinone biosynthesis protein D |
| PA1989 | *pqqE* | 2.72 | 2.77 |  |  | pyrroloquinoline quinone biosynthesis protein E |
| PA2014 | *liuB* | -2.25 | -2.67 |  |  | methylcrotonyl-CoA carboxylase. beta-subunit |
| PA2015 | *liuA* |  | -2.4 |  |  | putative isovaleryl-CoA dehydrogenase |
| PA2034 |  |  |  | -2.24 | -4.33 | hypothetical protein |
| PA2191 | *exoY* |  |  |  |  | adenylate cyclase ExoY |
| PA2193 | *hcnA* | -2.49 |  |  |  | hydrogen cyanide synthase |
| PA2194 | *hcnB* | -2.29 |  |  |  | hydrogen cyanide synthase |
| PA2195 | *hcnC* | -2.59 |  |  |  | hydrogen cyanide synthase |
| PA2223 |  | 2.16 |  |  |  | hypothetical protein |
| PA2247 | *bkdA1* | -2.36 | -3.81 |  |  | 2-oxoisovalerate dehydrogenase (alpha subunit) |
| PA2248 | *bkdA2* | -2.60 | -3.51 |  |  | 2-oxoisovalerate dehydrogenase (beta subunit) |
| PA2249 | *bkdB* | -2.48 | -3.22 |  |  | branched-chain alpha-keto acid dehydrogenase |
| PA2250 | *lpdV* | -2.62 | -3.07 |  |  | lipoamide dehydrogenase-Val |
| PA2264 |  | 2.63 |  |  |  | conserved hypothetical protein |
| PA2265 |  | 2.15 |  |  |  | gluconate dehydrogenase |
| PA2321 |  | 5.18 |  |  |  | gluconokinase |
| PA2322 |  | 3.77 |  |  |  | gluconate permease |
| PA2360 |  |  |  |  | -3.22 | hypothetical protein |
| PA2365 |  | 3.34 | 3.12 |  |  | conserved hypothetical protein |
| PA2366 |  | 2.72 | 2.38 |  |  | conserved hypothetical protein |
| PA2367 |  | 3.47 | 3.28 |  |  | hypothetical protein |
| PA2368 |  | 2.07 |  | -2.15 | -3.53 | hypothetical protein |
| PA2371 |  |  |  | -2.18 | -3.40 | probable ClpA/B-type protease |
| PA2372 |  |  |  | -2.09 | -2.77 | hypothetical protein |
| PA2381 |  | -3.08 |  |  |  | hypothetical protein |
| PA2390 | *pvdT* |  |  |  | -2.65 | PvdT. probable ATP-binding/permease fusion ABC transporter |
| PA2433 |  | 2.15 |  |  |  | hypothetical protein |
| PA2442 | *gcvT2* |  | 2.29 |  |  | glycine cleavage system protein T2 |
| PA2443 | *sdaA* |  | 2.74 |  |  | L-serine dehydratase |
| PA2444 | *glyA2* |  | 3.58 |  |  | serine hydroxy methyltransferase |
| PA2554 |  | -2.00 |  |  |  | probable short-chain dehydrogenase |
| PA2618 |  | 2.14 |  |  |  | hypothetical protein |
| PA2622 | *cspD* | 2.16 |  |  |  | cold-shock protein CspD |
| PA2566 |  |  |  | -2.89 | -4.26 | conserved hypothetical protein |
| PA2624 | *idh* |  |  | 2.54 | 3.16 | isocitrate dehydrogenase |
| PA2634 | *aceA* | 2.87 | 2.45 |  |  | isocitrate lyase AceA |
| PA2663 |  | 2.15 |  |  |  | hypothetical protein |
| PA2679 |  | 3.14 | 2.55 |  |  | hypothetical protein |
| PA2699 |  |  |  |  | -2.54 | hypothetical protein |
| PA2747 |  | 2.65 |  |  |  | hypothetical protein |
| PA2779 |  |  |  |  | -3.05 | hypothetical protein |
| PA2788 |  | -2.28 |  |  |  | probable chemotaxis transducer |
| PA2862 | *lipA* |  | 2.63 |  |  | lactonizing lipase precursor LipA |
| PA2939 |  | 3.29 | 2.47 | -3.42 | -4.76 | probable aminopeptidase |
| PA3038 |  | -4.13 |  | 2.11 |  | probable porin |
| PA3049 | *rmf* | 2.31 |  |  |  | ribosome modulation factor Rmf |
| PA3054 |  |  |  |  | 2.39 | hypothetical protein |
| PA3121 | *leuC* |  |  | 2.23 | 4.06 | 3-isopropylmalate dehydratase large subunit |
| PA3181 |  | 2.18 |  |  |  | 2-keto-3-deoxy-6-phosphogluconate aldolase |
| PA3183 | *zwf* | 2.43 |  |  |  | glucose-6-phosphate 1-dehydrogenase |
| PA3188 |  |  | -3.84 |  |  | probable permease of ABC sugar transporter |
| PA3189 |  |  | -2.05 |  |  | probable permease of ABC sugar transporter |
| PA3190 |  |  | -7.81 |  |  | probable binding protein component of ABC sugar transporter |
| PA3250 |  |  | 3.01 |  | -3.26 | hypothetical protein |
| PA3278 |  | 2.13 | 2.50 |  |  | hypothetical protein |
| PA3326 |  |  | 2.54 |  |  | probable Clp-family ATP-dependent protease |
| PA3328 |  |  | 2.80 |  |  | Probable FAD-dependent monooxygenase |
| PA3329 |  | 2.51 | 3.08 |  |  | hypothetical protein |
| PA3330 |  | 2.14 |  |  |  | probable short chain dehydrogenase |
| PA3331 |  | 2.37 | 3.00 |  |  | cytochrome P450 |
| PA3332 |  | 2.45 | 3.31 |  |  | conserved hypothetical protein |
| PA3333 | *fabH2* | 2.21 | 2.98 |  |  | 3-oxoacyl-[acyl-carrier-protein] synthase III |
| PA3334 |  | 3.28 | 3.75 |  |  | probable acyl carrier protein |
| PA3335 |  |  | 2.62 |  |  | hypothetical protein |
| PA3361 | *lecB* |  |  | -5.02 | -5.20 | fucose-binding lectin PA-IIL |
| PA3366 | *amiE* | 2.04 |  |  |  | aliphatic amidase |
| PA3392 | *nosZ* | -3.05 | -3.77 |  |  | nitrous-oxide reductase precursor |
| PA3393 | *nosD* | -2.18 | -2.11 |  |  | NosD protein |
| PA3442 |  |  |  |  | 2.05 | probable ATP-binding component of ABC transporter |
| PA3451 |  | 2.21 |  |  |  | hypothetical protein |
| PA3452 | *mqoA* | 3.12 |  |  |  | malate:quinone oxidoreductase |
| PA3465 |  | 2.05 | 2.34 |  |  | conserved hypothetical protein |
| PA3478 | *rhlB* | 2.46 | 3.98 | -2.45 | -4.71 | rhamnosyltransferase chain B |
| PA3479 | *rhlA* | 3.14 | 3.68 |  |  | rhamnosyltransferase chain A |
| PA3531 | *bfrB* | 2.17 |  |  |  | bacterioferritin |
| PA3578 |  |  |  |  | -2.17 | conserved hypothetical protein |
| PA3602 |  | 2.35 | 2.26 |  | 3.11 | conserved hypothetical protein |
| PA3613 |  |  |  |  | 2.65 | hypothetical protein |
| PA3614 |  | 2.02 | 2.14 |  |  | hypothetical protein |
| PA3684 |  | 2.06 |  |  |  | hypothetical protein |
| PA3688 |  | 2.38 | 2.28 |  |  | hypothetical protein |
| PA3691 |  | 2.10 |  |  |  | hypothetical protein |
| PA3723 |  | 2.43 | 2.15 |  |  | probable FMN oxidoreductase |
| PA3724 | *lasB* |  |  | -3.66 | -7.54 | elastase LasB |
| PA3735 | *thrC* |  |  |  | 2.06 | threonine synthase |
| PA3790 | *oprC* | -2.50 | -2.34 |  |  | outer membrane protein |
| PA3836 |  | -2.14 | -3.15 |  | -2.55 | hypothetical protein |
| PA3838 |  |  | -2.57 |  |  | probable ATP-binding component of ABC transporter |
| PA3841 | *exoS* | 2.07 |  |  | -4.27 | exoenzyme S |
| PA3842 | *spcS* |  |  |  | -3.28 | specific Pseudomonas chaperone for ExoS. SpcS |
| PA3843 |  |  |  |  | -2.35 | hypothetical protein |
| PA3858 |  |  |  |  | -2.13 | probable amino acid-binding protein |
| PA3866 |  |  |  | -2.03 | -3.70 | pyocin protein |
| PA3871 |  | -2.31 | -2.30 |  |  | probable peptidyl-prolyl cis-trans isomerase. PpiC-type |
| PA3872 | *narI* | -4.76 | -4.11 |  |  | respiratory nitrate reductase gamma chain |
| PA3873 | *narJ* | -4.48 | -4.31 |  |  | respiratory nitrate reductase delta chain |
| PA3874 | *narH* | -6.92 | -6.05 |  |  | respiratory nitrate reductase beta chain |
| PA3875 | *narG* | -4.46 | -4.79 |  |  | respiratory nitrate reductase alpha chain |
| PA3876 | *narK2* | -2.67 | -2.06 |  |  | nitrite extrusion protein 2 |
| PA3877 | *narK1* | -2.81 |  |  |  | nitrite extrusion protein 1 |
| PA3911 |  | -2.03 |  |  | 2.62 | conserved hypothetical protein |
| PA3912 |  | -2.75 |  |  | 2.62 | conserved hypothetical protein |
| PA3915 | *moaB1* | -5.83 | -3.64 |  |  | molybdopterin biosynthetic protein B1 |
| PA3921 |  |  |  |  | -2.00 | probable transcriptional regulator |
| PA3922 |  |  |  | -4.42 | -5.08 | conserved hypothetical protein |
| PA3923 |  |  |  | -5.30 | -5.79 | hypothetical protein |
| PA3924 |  |  |  | -2.22 | -3.36 | probable medium-chain acyl-CoA ligase |
| PA3935 | *tauD* |  |  |  | 2.47 | taurine dioxygenase |
| PA3972 |  |  | -2.34 |  |  | probable acyl-CoA dehydrogenase |
| PA4015 |  |  |  |  | -2.35 | conserved hypothetical protein |
| PA4129 |  | -2.94 |  |  |  | hypothetical protein |
| PA4130 |  | -3.33 |  |  |  | probable sulfite or nitrite reductase |
| PA4131 |  | -9.75 | -2.93 |  | 2.75 | probable iron-sulfur protein |
| PA4132 |  | -5.47 |  |  |  | conserved hypothetical protein |
| PA4133 |  | -8.80 | -3.03 |  |  | cytochrome c oxidase subunit (cbb3-type) |
| PA4134 |  | -3.05 |  |  |  | hypothetical protein |
| PA4139 |  |  |  | 2.87 | 4.72 | hypothetical protein |
| PA4141 |  |  | 3.01 |  |  | hypothetical protein |
| PA4209 | *phzM* |  |  | -2.67 | -3.37 | probable O-methyltransferase |
| PA4211 | *phzB1* |  |  | -8.53 | -18.27 | probable phenazine biosynthesis protein |
| PA4217 | *phzS* |  |  | -6.40 | -7.91 | flavin-containing monooxygenase |
| PA4220 |  |  |  | -2.70 | -3.38 | hypothetical protein |
| PA4235 | *bfrA* |  |  |  | 2.07 | bacterioferritin |
| PA4236 | *katA* |  |  | 2.86 | 4.32 | catalase |
| PA4296 | *pprB* |  |  |  | -3.20 | two-component response regulator. PprB |
| PA4306 | *flp* |  |  | -6.66 | -8.64 | Type IVb pilin. Flp |
| PA4328 |  |  |  | 2.10 | 3.01 | hypothetical protein |
| PA4333 |  |  |  | 2.78 | 5.35 | probable fumarase |
| PA4348 |  |  |  |  | 2.66 | conserved hypothetical protein |
| PA4366 | *sodB* |  |  | 2.69 | 4.51 | superoxide dismutase |
| PA4430 |  |  |  | 2.10 | 3.19 | probable cytochrome b |
| PA4431 |  |  |  | 2.17 | 3.94 | probable iron-sulfur protein |
| PA4496 |  | -2.54 | -2.55 | -2.73 | -8.18 | probable binding protein component of ABC transporter |
| PA4497 |  |  |  | -2.97 | -4.81 | probable binding protein component of ABC transporter |
| PA4500 |  | -3.29 | -3.94 |  |  | probable binding protein component of ABC transporter |
| PA4501 | *opdD* | -2.53 |  |  |  | glycine-glutamate dipeptide porin OpdP |
| PA4520 |  |  |  |  | -2.14 | probable chemotaxis transducer |
| PA4523 |  |  |  |  | -2.12 | hypothetical protein |
| PA4571 |  |  |  | 2.28 | 4.45 | probable cytochrome c |
| PA4587 | *ccpR* | -5.26 | -3.94 | 3.91 | 7.36 | cytochrome c551 peroxidase precursor |
| PA4607 |  | 3.80 | 3.08 |  |  | hypothetical protein |
| PA4614 | *mscL* | 2.21 |  |  |  | conductance mechanosensitive channel MscL |
| PA4619 |  | 3.51 | 2.48 |  |  | probable c-type hypothetical protein cytochrome |
| PA4620 |  | 3.12 |  |  |  | hypothetical protein |
| PA4621 |  | 2.00 |  |  |  | probable oxidoreductase |
| PA4661 |  |  | 2.20 |  |  | hypothetical protein |
| PA4683 |  |  |  |  | 2.63 | hypothetical protein |
| PA4695 | *ilvH* |  |  |  | 2.03 | acetolactate synthase isozyme III small subunit |
| PA4714 |  | -2.25 |  |  |  | conserved hypothetical protein |
| PA4733 | *acsB* |  |  |  | -2.66 | acetyl-coenzyme A synthetase |
| PA4738 |  | 5.57 | 3.97 |  |  | conserved hypothetical protein |
| PA4739 |  | 8.93 | 5.45 |  |  | conserved hypothetical protein |
| PA4773 |  |  |  |  | 2.12 | hypothetical protein |
| PA4811 | *fdnH* |  |  |  | 2.58 | nitrate-inducible formate dehydrogenase. beta subunit |
| PA4880 |  | 2.52 |  |  | 2.59 | probable bacterioferritin |
| PA4913 |  |  |  |  | -2.07 | probable binding protein component of ABC transporter |
| PA4919 | *pcnB1* |  | -2.07 |  |  | nicotinate phosphoribosyltranferase |
| PA5036 | *gltB* |  |  |  | 2.06 | glutamate synthase large chain precursor |
| PA5053 | *hslV* |  |  |  | 2.02 | heat shock protein HslV |
| PA5060 | *phaF* |  |  |  | -2.42 | polyhydroxyalkanoate synthesis protein PhaF |
| PA5112 | *estA* |  | -2.25 |  | -2.23 | esterase EstA |
| PA5153 |  | -2.43 | -2.55 |  | -2.28 | probable periplasmic binding protein |
| PA5167 | *dctP* | -4.35 | -4.05 |  | -6.85 | probable c4-dicarboxylate-binding protein |
| PA5168 | *dctQ* | -2.19 | -2.12 |  | -6.40 | probable dicarboxylate transporter |
| PA5169 | *dctM* | -2.41 | -2.20 |  | -10.69 | probable C4-dicarboxylate transporter |
| PA5208 |  |  | 2.11 |  |  | conserved hypothetical protein |
| PA5220 |  |  |  |  | -4.08 | hypothetical protein |
| PA5300 | *cycB* |  |  |  | 3.32 | cytochrome c5 |
| PA5348 |  |  |  |  | -3.03 | probable DNA-binding protein |
| PA5415 | *glyA1* |  | 2.28 |  |  | serine hydroxymethyltransferase |
| PA5445 |  | 2.57 | 2.97 | 2.30 | 4.67 | probable coenzyme A transferase |
| PA5446 |  | 2.67 |  | -3.22 | -3.58 | hypothetical protein |
| PA5482 |  | 2.13 |  |  |  | hypothetical protein |
| PA5546 |  | 2.28 |  |  | -2.46 | conserved hypothetical protein |
| PA5549 | *glmS* |  |  |  | 2.18 | glucosamine-fructose-6-phosphate aminotransferase |
