# Supplementary material for: Novel Targets of the CbrAB/Crc Carbon Catabolite Control System Revealed by Transcript Abundance in Pseudomonas aeruginosa
Source: PLoS One. 2012 Oct 24;7(10):e44637. doi: 10.1371/journal.pone.0044637 (PMC3480352; doi:10.1371/journal.pone.0044637)
Supplement: Table S3 — Strains and plasmids used in this study. (DOC) [file pone.0044637.s005.doc]

**Table S3.** Strains and plasmids used in this study

| **Strains/plasmid** | **Genotype/relevant features** | **Source/reference^a^** |
| --- | --- | --- |
|  |  |  |
| ***P. aeruginosa*** |  |  |
| PAO1 | Wild type | [46] |
| PAO6673 | Δ*crc* | [1] |
| PAO6679 | Δ*crcZ* promoter | [1] |
| PAO6711 | Δ*cbrB* | [1] |
|  |  |  |
| ***E. coli*** |  |  |
| BL21(DE3) | F^–^ *ompT hsdS_B_*(r_B_-, m_B_-) *dcm gal* λ(DE3) | Novagen |
| DH5α | *recA1 endA1 hsdR17 thi-1 supE44 gyrA96 relA1 deoR* Δ(*lacZYA-argF*) *U169* (Φ80*lacZ*ΔM15) Nal^r^ | [47] |
| XL1-Blue | *recA1 endA1 gyrA96 thi-1 hsdR17*(r_K_-, m_K_+) *supE44 relA1 lac* [F' *proAB* *lacI^q^Z*ΔM15::Tn*10*(Tc^r^)] | Stratagene |
|  |  |  |
| **Plasmids** |  |  |
| pME6015 | Cloning vector for translational *lacZ* fusions; Tc^r^ | [48] |
| pME9655 | Plasmid carrying a translational *amiE’-‘lacZ* fusion | [1] |
| pME10044 | pME6015 derivative carrying a translational *acsA’-‘lacZ* fusion | This study |
| pME10045 | pME10044 where the CA-motif AACAAAAACAA of *acsA* was replaced by TGATCAGTAGC | This study |
| pME10046 | pME6015 derivative carrying a translational *aroP2’-‘lacZ* fusion | This study |
| pME10047 | pME10046 where the CA-motif AACAATAA of *aroP2* was replaced by TCAGTAGC | This study |
| pME10048 | pME6015 derivative carrying a translational *bkdR’-‘lacZ* fusion | This study |
| pME10049 | pME6015 derivative carrying a translational *bkdA’-‘lacZ* fusion | This study |
| pTL*estA* | pME6015 derivative carrying a translational *estA’-‘lacZ* fusion | This study |
| pTL*estA*-ΔCA | pTLestA where the CA-motif AAAAACAA of *estA* was replaced by TCAGTAGC | This study |
|  |  |  |
|  |  |  |

^a^ Additional references:

46. Holloway BW, Krishnapillai V, Morgan AF (1979) Chromosomal genetics of *Pseudomonas*. Microbiol Rev 43: 73-102.

47. Sambrook J, Russell DW (2001) Molecular Cloning: A Laboratory Manual. Cold Spring Harbour: Cold Spring Harbor Laboratory Press.

48. Schnider-Keel U, Seematter A, Maurhofer M, Blumer C, Duffy B, Gigot-Bonnefoy C, Reimmann C, Notz R, Défago G, Haas D, Keel C (2000) Autoinduction of 2,4-diacetylphloroglucinol biosynthesis in the biocontrol agent *Pseudomonas fluorescens* CHA0 and repression by the bacterial metabolites salicylate and pyoluteorin. J Bacteriol 182: 1215-1225.
