# Supplementary material for: Novel Targets of the CbrAB/Crc Carbon Catabolite Control System Revealed by Transcript Abundance in Pseudomonas aeruginosa
Source: PLoS One. 2012 Oct 24;7(10):e44637. doi: 10.1371/journal.pone.0044637 (PMC3480352; doi:10.1371/journal.pone.0044637)
Supplement: Table S4 — DNA oligonucleotides used in this study. (DOC) [file pone.0044637.s006.doc]

**Table S4.** DNA oligonucleotides used in this study

| Name | Sequence^a^ (5’ → 3’) | Restriction site |
| --- | --- | --- |
| SDM-CAtrap1 | CCCGTCCTCTTCATAAAAGACAGACTCAGTAGCAGAGGAAGTCTC |  |
| SDM-CAtrap2 | GAGACTTCCTCTGCTACTGAGTCTGTCTTTTATGAAGAGGACGGG |  |
| AcsA1 | G**GAATTC**AGGTCGCGGCGGCGGGGCG | *Eco*RI |
| AcsA2 | CCAATGCATTGGTT**CTGCAG**GGATGCCGCAGACATGGCTTTAACC | *Pst*I |
| AroP21 | G**GAATTC**ATCCCGCTGCCGCCAGCCTCTTCCG | *Eco*RI |
| AroP22 | CCAATGCATTGGTT**CTGCAG**CGGATGTTGGCCGTCCATTGAATTATCTCACGC | *Pst*I |
| AcsAmutfw | CGCTACAACTGGGCACTGATCAGTAGCCACTCACGTACCGAGG |  |
| AcsAmutrev | CCTCGGTACGTGAGTGGCTACTGATCAGTGCCCAGTTGTAGCG |  |
| AroP2mutfw | CGTTTCGCCTCCGACTCAGTAGCATTTCCACTTGCGTG |  |
| AroP2mutrev | GACGCAAGTGGAAATGCTACTGAGTCGGAGGCGAAACG |  |
| PA2246fw | ACGT**GAATTC**CGTAATCACTCATGACAGGC | *Eco*RI |
| PA2246rev | ACGT**GGATCC**GAGTTCAGCCATGGTGCGTTG | *Bam*HI |
| PA2247fw | ACGT**GAATTC**CAGGTCGATGCGGTCGAG | *Eco*RI |
| PA2247rev | ACGT**GGATCC**GTAATCACTCATGACAGGC | *Bam*HI |
| Q67_estAfw | TTTTT**GAATTC**GAGCAGCCTGGCACGC | *Eco*RI |
| R67_estArev | TTTT**GGATCC**GAGCGCCATTCTGATCAT | *Bam*HI |
| L71_estAmut1a | GACGCGGCGGCCCGACAATtcagtagcATCATGGAGTAAG |  |
| M71_estAmut1b | CTTACTCCATGATGCTACTGAATTGTCGGGCCGCCGCGTC |  |
| EstAfw | GACTTCGACTCCCAGGACAG |  |
| EstArev | TGGCCATGTAGCTGTTCATC |  |
| AroP2fw | CGGTGGTGGTGAACTATGTG |  |
| AroP2rev | AGTTGCCGTACGGATACCAG |  |
| BkdRfw | GCATCGACCTGAAAATCCTC |  |
| BkdRrev | GTCTGCTTTTCCAGGGACAC |  |
| BkdA1fw | GCCTACAGCCTGGTACGTGT |  |
| BkdA1rev | GAAAGCTTTTTCTGCCGTTG |  |
| AcsAfw | GATTGCAAGTCGAAGGTGGT |  |
| AcsArev | GCTGGTTCCACTTGATCTCC |  |
| IlvEfw | GTGTTCTACGGAAGCGAAGG |  |
| IlvErev | GCCAGCATCGAGTTGATGTA |  |
| Flpfw | GAACCTGACCCTGTTCGTGT |  |
| Flprev | CGTCGAAGAAGGCTTTCAG |  |
| Anrfw | CACCTTCCTGGTCAACCTGT |  |
| Anrrev | CTCGATGGAGTCGAGGATGT |  |

^a^Restriction sites are highlighted in boldface and the CA-motif mutations are boxed.
